# Urinary Biomarkers of Exposure to Volatile Organic Compounds from the Population Assessment of Tobacco and Health Study Wave 1 (2013–2014)

**DOI:** 10.3390/ijerph17155408

**Published:** 2020-07-28

**Authors:** Víctor R. De Jesús, Deepak Bhandari, Luyu Zhang, Christopher Reese, Kimberly Capella, Denise Tevis, Wanzhe Zhu, Arseima Y. Del Valle-Pinero, Guy Lagaud, Joanne T. Chang, Dana van Bemmel, Heather L. Kimmel, Eva Sharma, Maciej L. Goniewicz, Andrew Hyland, Benjamin C. Blount

**Affiliations:** 1 Division of Laboratory Sciences, National Center for Environmental Health, Centers for Disease Control & Prevention, Atlanta, GA 30341, USA; xwo1@cdc.gov (D.B.); pkf7@cdc.gov (L.Z.); wri5@cdc.gov (C.R.); xhj9@cdc.gov (K.C.); iop5@cdc.gov (D.T.); poq5@cdc.gov (W.Z.); bkb3@cdc.gov (B.C.B.); 2 Office of Science, Center for Tobacco Products, Food and Drug Administration, Silver Spring, MD 20993, USA; Arseima.Reissig@fda.hhs.gov (A.Y.D.V.-P.); Guy.Lagaud@fda.hhs.gov (G.L.); Joanne.Chang@fda.hhs.gov (J.T.C.); Dana.vanBemmel@fda.hhs.gov (D.v.B.); 3 National Institute on Drug Abuse, National Institutes of Health, Bethesda, MD 20892, USA; heather.kimmel@nih.gov; 4 Westat, 1600 Research Boulevard, Rockville, MD 20850, USA; EvaSharma@westat.com; 5 Division of Cancer Prevention and Population Sciences, Roswell Park Comprehensive Cancer Center, Elm and Carlton Streets, Buffalo, NY 14263, USA; Maciej.Goniewicz@RoswellPark.org (M.L.G.); Andrew.Hyland@RoswellPark.org (A.H.)

**Keywords:** volatile organic compound metabolites, PATH Study, tobacco smoke exposure, e-cigarette users, smokeless tobacco users

## Abstract

Volatile organic compounds (VOCs) are ubiquitous in the environment. In the United States (U.S.), tobacco smoke is the major non-occupational source of exposure to many harmful VOCs. Exposure to VOCs can be assessed by measuring their urinary metabolites (VOCMs). The Population Assessment of Tobacco and Health (PATH) Study is a U.S. national longitudinal study of tobacco use in the adult and youth civilian non-institutionalized population. We measured 20 VOCMs in urine specimens from a subsample of adults in Wave 1 (W1) (2013–2014) to characterize VOC exposures among tobacco product users and non-users. We calculated weighted geometric means (GMs) and percentiles of each VOCM for exclusive combustible product users (smokers), exclusive electronic cigarette (e-cigarette) users, exclusive smokeless product users, and tobacco product never users. We produced linear regression models for six VOCMs with sex, age, race, and tobacco user group as predictor variables. Creatinine-ratioed levels of VOCMs from exposure to acrolein, crotonaldehyde, isoprene, acrylonitrile, and 1,3-butadiene were significantly higher in smokers than in never users. Small differences of VOCM levels among exclusive e-cigarette users and smokeless users were observed when compared to never users. Smokers showed higher VOCM concentrations than e-cigarette, smokeless, and never users. Urinary VOC metabolites are useful biomarkers of exposure to harmful VOCs.

## 1. Introduction

Volatile organic compounds (VOCs) are ubiquitous in the environment. In the United States (U.S.), tobacco smoke is the major non-occupational source of exposure to many harmful VOCs [1]. Tobacco smoke contains over 8000 chemicals, including carcinogenic and toxic VOCs such as acrylonitrile and benzene [2]. Lower levels of VOCs have been reported in electronic cigarette (e-cigarette) refill solutions and emissions; e-cigarette use has increased significantly worldwide, especially among youth [3,4]. Regardless of exposure source, high levels of harmful VOCs are a significant public health concern. Previous epidemiologic studies have suggested respiratory effects of VOC exposure (e.g., asthma exacerbation, lung cancer) [5,6,7]. Moreover, controlled human exposure studies have suggested the inflammatory effects of VOCs (i.e., dose-related increases in lower and upper respiratory symptoms) [8,9].

Exposure to VOCs can be assessed by measuring their metabolites in urine. While measuring VOCs in blood can provide a direct assessment of VOC levels in vivo [1,10], urinary biomarkers of VOC exposure have a longer biological half-life than VOCs in blood and are more stable during storage and handling [11]. Volatile organic compound metabolites (VOCMs) have been examined in the U.S. population through the National Health and Nutrition Examination Survey (NHANES) since 2005 [12,13]. However, the NHANES is not designed to address tobacco-related exposures specifically, and thus includes relatively few users of tobacco products other than cigarettes. Population data are needed to better evaluate harmful VOC exposures resulting from use of various tobacco products.

The Population Assessment of Tobacco and Health (PATH) Study is a U.S. cohort study of 45,971 adults and youth, aged 12 years or older, that studies tobacco use and its health effects in the population [14]. In Wave 1 (W1) of the study, we measured VOCMs in urine specimens collected from adult tobacco users and non-users to characterize and compare VOC exposures among tobacco users and non-users in the U.S. The primary aim of the study was to estimate urinary concentrations of 20 VOCMs stratified by four user groups (e.g., Every day established exclusive combustible product user, Every day established exclusive e-cigarette user, Every day established exclusive smokeless product user, and never user). The measurement of urinary biomarkers of VOC exposure will help characterize the prevalence and magnitude of exposure to tobacco-related VOCs.

## 2. Materials and Methods

### 2.1. Study Design

We used biomarker and questionnaire data from W1 of the PATH Study, conducted from September 12, 2013 to December 15, 2014. Adult tobacco users, young adults aged 18 to 24, and African Americans were oversampled relative to population proportions. The weighting procedures were adjusted for oversampling and nonresponse. Combined with the use of a probability sample, the weighted data allowed the estimates produced by the PATH Study to be representative of the non-institutionalized, civilian U.S. population. Further details regarding the PATH Study design and methods are published elsewhere [14]. Details on survey interview procedures, questionnaires, sampling, weighting, and information on accessing the data are available at https://doi.org/10.3886/Series606. The Westat Institutional Review Board approved the study design and data collection protocol.

### 2.2. Participants

Participants completed the W1 adult interview and provided detailed information about their tobacco product use (i.e., self-reported). Our analyses focused on the following four mutually exclusive tobacco use categories: (1) Every Day Established Exclusive Combustible Product User (Smokers); (2) Every Day Established Exclusive E-cigarette User (electronic nicotine delivery system (ENDS) Users); (3) Every Day Established Exclusive Smokeless Product User (Smokeless Users); and (4) Never Users of Tobacco (Never Users) (see Table 1). In these analyses, tobacco products are defined as follows: combustible products, which include cigarettes, cigars, cigarillos, little filtered cigars, pipe, and hookah; e-cigarettes; and smokeless tobacco products, which include loose snus, pouched snus, chewing tobacco, dip, snuff, spit tobacco, and dissolvable tobacco.

### 2.3. Biospecimen Collection Procedures

Full-void spot urine specimens from 11,501 consenting participants were self-collected in a 500 mL polypropylene container (Globe Scientific, Mahwah, NJ, USA), immediately placed in a Crēdo Cube shipper (Series 4–496, Minnesota Thermal Science, Plymouth, MN, USA) certified to hold contents 2–8 °C for at least 72 h, and shipped overnight to the PATH Study biorepository. Each specimen was divided into aliquots and stored in FluidX^®^ (Brooks Life Sciences, Chelmsford, MA, USA) polypropylene cryovials at −80 °C. Study participants used tobacco products ad libitum during the in-home interview, without controlling the timing of last tobacco use and spot urine collection.

### 2.4. Chemical Analysis

#### 2.4.1. Volatile Organic Compound Metabolites (VOCMs)

In all, 20 biomarkers of exposure to VOCs (Table 2) were measured in spot urine using isotope dilution UPLC-MS/MS as described by Alwis et al. [15] and modified by Alwis et al. [16]. Briefly, samples were assayed by analyzing a 50 µL aliquot of each specimen through an ultra-high-performance liquid chromatography system (Waters Inc., Milford, MA, USA) coupled with electrospray ionization (ESI) tandem mass spectrometry (Sciex API 5500 Triple Quad, Applied Biosystems, Foster City, CA, USA). The mass spectrometer was operated using negative-ion ESI and in scheduled multiple reaction monitoring mode. The ion source temperature was held at 650 °C, and the electrospray ion voltage was −4000 V. Urine specimens were assayed with a 1:10 dilution (50 µL urine +25 µL mixed internal standard +425 µL 15 mM ammonium acetate). The mobile phase consisted of 15 mM ammonium acetate, pH 6.8 (mobile phase A), and acetonitrile (mobile phase B). Unknown concentrations were determined using the peak area ratio of a known standard to the stable isotope-labeled internal standard. The limits of detection (LODs) ranged from 0.5 ng/mL to 15 ng/mL. The reported data meet the quality requirements of the Centers for Disease Control and Prevention (CDC) Division of Laboratory Sciences [17].

#### 2.4.2. Urinary Creatinine

Urinary biomarker concentrations can be influenced by urine dilution, which can vary markedly from void to void and may confound statistical inference [18]. Urine dilution can be accounted for by scaling urinary analyte concentration to the urinary concentration of creatinine, a compound formed endogenously by lean body mass and excreted at a fairly constant rate. Creatinine-corrected values were calculated for urinary biomarkers of respondents with levels of creatinine between 10 and 370 mg/dL. This correction helped to avoid the confounding effects of overly dilute or hyper-concentrated urines [19]. Creatinine in urine was measured by an enzymatic assay on a commercial automated clinical chemistry analyzer, and the LOD was 1.1 mg/dL.

## 3. Statistical Analysis

The PATH Study recruited participants through a multistage probability sample. Applying survey weights produced robust estimates. Balanced repeated replication (BRR) with a Fay’s adjustment factor of 0.3 was used for variance estimation. We used this estimation approach as implemented in the PROC DESCRIPT, PROC CROSSTAB, and PROC VARGEN procedures of SUDAAN version 11.0.1 (Research Triangle Institute, Research Triangle Park, NC, USA) called from the SAS statistical software application version 9.4 (SAS Institute, Cary, NC, USA). Statistical significance was set to 0.05.

A total of 11,501 samples with VOCM data were retrieved from PATH Study W1 data. Of those, 4075 people were excluded because of missing demographic variables, 2049 people were excluded for dual/poly product use, and an additional 128 people were excluded for missing creatinine measurements (i.e., creatinine-ratioed VOCM values were used for all descriptive statistics). Furthermore, 28 people were excluded if they reported smoking >100 cigarettes per day. Because the distribution of measurements was strongly right-skewed, VOCM concentrations were log-transformed to calculate the weighted geometric mean (GM) of each VOCM by user group, and the weighted percentile (e.g., 5th, 10th, 25th, 50th, 75th, 90th, and 95th percentile) numerical distribution of the data for each VOCM by user group.

### 3.1. Multivariate Analysis and Selected VOCM Analyses

We investigated the effect of selected predictors on VOCM levels by tobacco user group. Sample-weighted multivariate linear regression models stratified were fit to W1 data, where the dependent variables were urinary concentrations (ng/mL) of six VOCMs (2COEMA, 2CYEMA, 3HPMA, 3HMPMA, 4HMBEMA, and t4HBEMA). Sex, age, race/ethnicity, education, tobacco user group, and creatinine concentration were examined as predictors in each model. These six VOCMs were selected because their parent VOCs (acrolein, acrylonitrile, crotonaldehyde, isoprene, and 1,3-butadiene) have high cancer and non-cancer risk indices based on exposure to a single cigarette per day [20]. Moreover, they have been prioritized by the World Health Organization as part of a strategy for tobacco regulation based on product performance assessments, with the goal of reducing the mainstream smoke levels of selected constituents [21,22]. The selected VOCMs are also included in the Food and Drug Administration (FDA)’s Established List of Harmful and Potentially Harmful Constituents in Tobacco Products and Tobacco Smoke [23].

Because the distribution of measurements was strongly right-skewed, which would have adversely affected hypothesis testing, urinary VOCM concentration data were natural log-transformed for regression analysis. We report coefficients from these models along with their 95% confidence intervals and *p*-values. To interpret categorical predictors in the model, if the exponentiated coefficient estimate is greater or less than 1, the predictive percentage increase or decrease in VOCM concentrations is calculated as the estimate minus one then multiplied by 100.

### 3.2. PATH Study W1 and NHANES 2015–2016 VOCM Comparison

VOCM data from the PATH Study Wave 1 (2013–2014) for six VOCMs (2COEMA, 2CYEMA, 3HPMA, 3HMPMA, 4HMBEMA, and t4HBEMA) were compared with results from the NHANES 2015–2016 (UVOCS_I). The NHANES is a population-based survey designed to assess health and nutritional status through a cross-sectional observation of a complex, multistage probability sample representative of the civilian, non-institutionalized population of the United States [12]. Participants were classified as smokers and non-users as previously described [13,24,25]. Only smokers (serum cotinine > 10 ng/mL) and non-users (serum cotinine ≤ 10 ng/mL) were examined in this comparison since there were not enough NHANES study participants who could be apportioned into the other two user groups studied in this report. Only NHANES study participants 18 years or older were selected. In order to directly compare with NHANES participants, PATH Study Wave 1 participants were also classified as smokers and non-users based on their serum cotinine level. We performed two-sample *t*-tests on weight-adjusted samples to determine whether these two studies had equal GMs for the same analytes used in the multivariate analysis. Bonferroni correction was used to adjust for multiple comparisons to avoid the inflation of type I error.

## 4. Results

### 4.1. VOCM Geometric Means and Percentiles by Tobacco User Group

**Figure 1:**
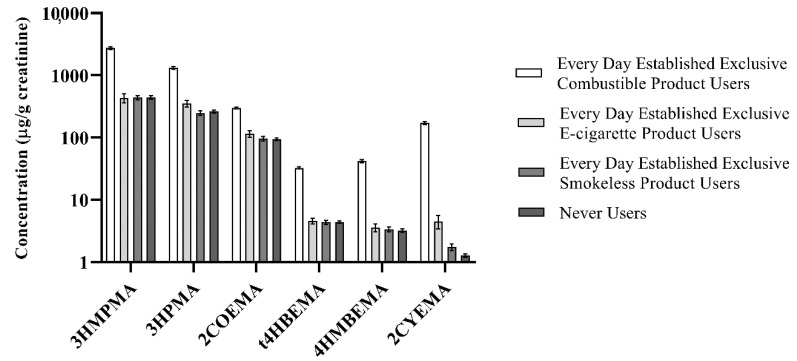
Creatinine-ratioed sample-weighted geometric means (GMs) (95% confidence interval) of selected VOCMs by tobacco user group in the PATH Study Wave 1 (2013–2014).

We examined 5221 subjects, after excluding participants who could not be assigned to any of the four abovementioned user groups. The sample size for each tobacco user group was as follows: (1) Smokers, *n =* 3156; (2) ENDS Users, *n* = 149; (3) Smokeless Users, *n* = 353; and (4) Never Users, *n =* 1563. Overall detection frequencies for 10 VOCMs were >99%; the detection frequency was lowest for 1CYHEMA (35.98%; Table S1, Supplementary Materials). We calculated creatinine- and non-creatinine-ratioed GMs by demographic and tobacco user group for 20 VOCMs (Tables S2–S21, Supplementary Materials). In addition, selected percentiles by tobacco user groups (both creatinine- and non-creatinine-ratioed) are provided in Tables S22–S41. Figure 1 presents GMs (95% confidence intervals) of six VOCMs by specific tobacco product user groups as described above. Concentrations of three biomarkers (BZMA, PHMA, and TTCA) were similar between all four tobacco user groups.

Overall, the relative abundance of VOCM GMs by tobacco user group follows the same pattern, i.e., Smokers had the highest VOCM GMs, followed by ENDS Users. Smokeless and Never Users had the lowest GMs out of the four groups and were about the same for most VOCMs. Of note, statistically significant differences between all four tobacco user group GMs were observed only for 2CYEMA and 3HPMA levels when compared to the reference group. Creatinine-ratioed 2CYEMA GMs (standard error) were as follows (µg/g creatinine): 172 (4.77) (Smokers), 4.51 (0.560) (ENDS), 1.76 (0.114) (Smokeless), and 1.27 (0.043) (Never Users). For 3HPMA, creatinine-ratioed GMs (standard error) were as follows (µg/g creatinine): 1.32 × 10^3^ (33.0) (Smokers), 354 (22.3) (ENDS), 249 (10.1) (Smokeless), and 262 (7.66) (Never Users).

### 4.2. Multivariate Analysis of Tobacco Smoke and Demographic Associations with Selected VOCMs

We found significantly higher (*p* < 0.0001) VOCM levels between Smokers and Never Users for 2COEMA (206%), 2CYEMA (>12,000%), 3HPMA (385%), 3HMPMA (505%), 4HMBEMA (1193%), and t4HBEMA (617%) after adjusting for age, sex, race, education level, and creatinine (Tables S42–S47). Contrastingly, race/ethnicity and age variables were associated with smaller magnitude changes in VOCM levels (Table 3). A positive number indicates significantly higher (*p* < 0.05) levels of VOCMs when compared to the reference group. Alternatively, a negative number indicates significantly lower levels of VOCMs when compared to the reference group. After controlling for other cofactors, females had significantly higher urinary 2COEMA, 3HMPMA, 4HMBEMA, and t4HBEMA levels compared to males. Likewise, after controlling for tobacco use and other cofactors and using participants’ age of 25–34 years as the reference, young adults (18–24 years) had significantly lower urinary concentrations of 2COEMA, 3HPMA, 3HMPMA, 4HMBEMA, and t4HBEMA (*p*-values <0.0009), and older adults (≥ 55 years) had statistically significantly higher urinary concentrations for the same five VOCMs (range 17.2–35.5%; *p*-values < 0.0008). In both cases, 2CYEMA levels showed no statistically significant changes among age groups.

### 4.3. PATH Study W1 and NHANES 2015–2016 VOCM Comparison

We compared weighted GMs (ng/mL) of selected VOCMs (2COEMA, 2CYEMA, 4HMBEMA, 3HMPMA, 3HPMA, and t4HBEMA) in the PATH Study Wave 1 and the NHANES 2015–2016 for Never Users and Smokers (as defined in previous sections) (Table 4). After adjusting for multiple comparisons, we observed small, but statistically significant, differences in GM levels for 3HPMA, 2COEMA, and 3HMPMA in Smokers (*p*-values < 0.0083, PATH > NHANES). For Smokers, 2CYEMA GMs were not statistically different between PATH Study Wave 1 and NHANES 2015–2016 participants (68.4 and 58.0 ng/mL, respectively); the same pattern was observed for 4HMBEMA. Additionally, we observed small, but statistically significant, differences in GM levels for t4HBEMA, 3HPMA, and 3HMPMA in Never Users (*p*-values < 0.0009, PATH > NHANES). Like Smokers, 2CYEMA and 4HMBEMA GMs were not statistically different between Never Users in PATH Study Wave 1 and NHANES 2015–2016 participants (1.25 and 1.41 ng/mL, and 3.14 and 3.22 ng/mL, respectively).

## 5. Discussion

We quantified 20 VOCMs in urine samples collected as part of the PATH Study Wave 1 (2013–2014) and present herein these data as U.S. reference values for different types of tobacco product users. We detected at least one of the 20 VOCMs in over 99% of sampled participants, confirming widespread exposure to harmful VOCs in the general U.S. adult population. This finding is consistent with data from the NHANES [1,13,24].

The creatinine-ratioed geometric means of 17 VOCMs followed the same pattern: Smokers > ENDS Users > Smokeless Users > Never Users. Specifically, the use of combustible tobacco products was associated with higher levels of selected metabolites of acrolein, acrylamide, acrylonitrile, 1,3-butadiene, crotonaldehyde, cyanide, N,N-dimethylformamide/methyl isocyanate, ethylbenzene/styrene, isoprene, propylene oxide, styrene, *o*-xylene, and *m,p*-xylene. This pattern is consistent with data from the NHANES and other previous reports [26,27,28,29]. In addition, we present creatinine-ratioed percentiles for each VOCM by tobacco user group to describe VOCM concentration distributions. Females generally showed higher creatinine-ratioed VOCM GM levels compared to males regardless of the tobacco user group classification, with only a few exceptions (e.g., 2COEMA ENDS male and female users had the same GMs). Non-Hispanic Whites had the highest GMs across all tobacco user groups with some exceptions. However, those exceptions should be interpreted with caution because they have low statistical precision. They are based on a sample size of less than 50 (in each case), or the coefficient of variation of the estimate is larger than 30%.

We investigated the effect of selected predictors on VOCM levels by tobacco user group. Levels of all six modeled VOCMs were significantly higher (*p* < 0.0001) in Smokers when compared to Never Users; the percent increase attributable to smoking was 2COEMA (206%), 3HPMA (385%), 3HMPMA (505%), t4HBEMA (617%), 4HMBEMA (1193%), and 2CYEMA (> 12,000%). These higher exposures are understandable based on the microgram quantities of the parent VOCs in the mainstream smoke from a single cigarette [30]. The 120-fold higher levels of 2CYEMA in smokers compared with non-users underscores the value of 2CYEMA as a selective smoke exposure biomarker. Interestingly, regression models also indicate that urinary 3HPMA and 2CYEMA were marginally higher in ENDS Users than Never Users, possibly because of higher secondhand smoke exposure and/or occasionally unreported smoking in ENDS users (many of whom are former smokers). We also observed differences in some VOCM levels by sex, after controlling for confounders. These differences in VOCM levels in females may be partially explained by creatinine adjustments. While urinary creatinine excretion is relatively consistent in an individual, the amount excreted can vary significantly between individuals based on lean body mass and other physiological factors. Creatinine production tends to be higher in males compared to females and higher in non-Hispanic Blacks compared with other races [18]. Additionally, VOCM levels differed modestly by age and race/ethnicity, after controlling for confounders. The predicted effect size for these demographic variables was consistently small across all age and race/ethnicity categories (Table 3) compared with tobacco smoke. These data confirm that tobacco smoke exposure is a more impactful determinant of VOCM levels than are demographic variables.

PATH Study W1 GMs for 2COEMA, 2CYEMA, 3HPMA, 3HMPMA, 4HMBEMA, and t4HBEMA were marginally higher in magnitude compared to those for NHANES 2015–2016 participants (4 of 6 were statistically higher). The demographic characteristics for PATH Study W1 and NHANES 2015–2016 participants were very similar, notwithstanding the difference in the number of participants in each study. The higher levels of smoke-related VOCM biomarkers may result from a combination of two factors: (1) the PATH Study selects participants based on tobacco use and thus may include heavier smokers than those of the NHANES, and (2) the PATH Study’s urine collection is likely sooner after last tobacco product use compared with that of the NHANES. In both studies, the GM ratio of 2CYEMA in Smokers/Never Users exceeded 40:1 despite the fact that we did not exclude non-users who have secondhand smoke exposure. The consistency of higher urinary 2CYEMA in smokers across both studies provides further evidence for the use of 2CYEMA as a smoke exposure biomarker.

There are some important limitations to our study. Notably, we did not evaluate dual/poly use in our report since other studies have reported dual/poly use analyses using PATH Study Wave 1 (2013–2014) data [29]. The analyses performed by Goniewicz et al. are focused on characterizing harmful and addictive exposures in dual users of cigarettes and e-cigarettes. Additionally, other studies also characterized tobacco exposure biomarkers (including VOCs) in other combinations of tobacco product use [31]. Of note, we measured biomarkers of exposure to 20 different VOCs. However, we did not assess exposure to formaldehyde, which is a known human carcinogen found in emissions from some ENDS products [32]. Another limitation is that the PATH Study W1 captures data on first generation e-cigarettes that differ in product characteristics and operational features compared to e-cigarette products on the market currently. Our report describes the users of first-generation e-cigarettes [33], which were not as common as they are today (*n* = 149 for our analysis). Efforts are currently underway to characterize exposures related to the use of nicotine salt e-cigarettes in more recent waves of both the PATH Study and the NHANES. These analyses will be able to reference our current study as a baseline measure. We believe that it is important to document VOC exposures in the U.S. population at different times, and thus better understand the impact of changes in products, behaviors, and regulations.

## 6. Conclusions

We present the first comprehensive VOC exposure data in users of combustible tobacco products, e-cigarettes, smokeless tobacco products, and never users during the PATH Study Wave 1 (2013–2014), based on the analysis of 20 urinary biomarkers of exposure. Users of combustible tobacco products had significantly higher concentrations of most VOCMs than users of non-combustible tobacco products and never users. Users of smokeless tobacco products and e-cigarette users have VOC exposure similar to never users for most VOCMs, but not for 2CYEMA. Our findings suggest that selected VOCMs are suitable biomarkers for monitoring tobacco smoke exposure. Specifically, the biomarker 2CYEMA (its parent compound is acrylonitrile) was shown to effectively distinguish between combustible and non-combustible tobacco product users, and never users. The data presented in this study establish a baseline of exposures to VOCs to identify exposure trends resulting from changes in tobacco products (e.g., the emergence of nicotine salt e-cigarettes) and use patterns.

## Figures and Tables

**Table 1:** Tobacco user group definitions.

Tobacco User Group	Definition
Every Day Established Combustible Product User (Smokers; *n* = 3156)	For at least one combustible product: Has ever used the product If cigarettes, has smoked 100 cigarettes in lifetime. If other product, has used the product fairly regularly Currently uses every day Does not now use any smokeless products (answer “No” for each smokeless product questions from questionnaire) or e-cigarettes AND are not currently using any nicotine replacement therapy (NRT).
Every Day Established E-cigarette User (ENDS Users; *n* = 149)	Respondents who have used e-cigarettes every day AND are not current established or experimental users of any combustible product or smokeless tobacco, AND are not currently using any nicotine replacement therapy (NRT).
Every Day Established Smokeless Product User (Smokeless Users; *n* = 353)	For at least one smokeless tobacco product: Has ever used the product Has used the product fairly regularly Currently uses every day Does not now use any combustible products nor e-cigarettes AND are not currently using any nicotine replacement therapy (NRT).
Never Users (*n* = 1563)	Has never used any tobacco product.

**Table 2:** Urinary volatile organic compound metabolites (VOCMs) measured for the Population Assessment of Tobacco and Health (PATH) Study Wave 1 (W1).

Biomarker	Abbreviation	Parent Compound
*N*-Acetyl-S-(4-hydroxy-2-buten-1-yl)-L-cysteine	t4HBEMA	1,3-Butadiene
*N*-Acetyl-S-(3,4-dihydroxybutyl)-L-cysteine	34HBMA	
*N*-Acetyl-S-(3-hydroxypropyl)-L-cysteine	3HPMA	Acrolein
*N*-Acetyl-S-(2-carboxyethyl)-L-cysteine	2COEMA	
*N*-Acetyl-S-(2-carbamoylethyl)-L-cysteine	2CAEMA	Acrylamide
*N*-Acetyl-S-(2-carbamoyl-2-hydroxyethyl)-L-cysteine	2CAHEMA	
*N*-Acetyl-S-(2-cyanoethyl)-L-cysteine	2CYEMA	Acrylonitrile
*N*-Acetyl-S-(1-cyano-2-hydroxyethyl)-L-cysteine	1CYHEMA	
*N*-Acetyl-S-(2-hydroxyethyl)-L-cysteine	2HEMA	Acrylonitrile; Vinyl chloride; Ethylene oxide
*N*-Acetyl-S-(phenyl)-L-cysteine	PHMA	Benzene
2-Thioxothiazolidine-4-carboxylic acid	TTCA	Carbon disulfide
*N*-Acetyl-S-(3-hydroxypropyl-1-methyl)-L-cysteine	3HMPMA	Crotonaldehyde
*N*-Acetyl-S-(N-methylcarbamoyl)-L-cysteine	MCAMA	N,N-Dimethylformamide; Methyl isocyanate
Phenylglyoxylic acid	PHGA	Ethylbenzene; Styrene
*N*-Acetyl-S-(4-hydroxy-2-methyl-2-buten-1-yl)-L-cysteine	4HMBEMA	Isoprene
*N*-Acetyl-S-(2-hydroxypropyl)-L-cysteine	2HPMA	Propylene oxide
Mandelic acid	MADA	Ethylbenzene; Styrene
*N*-Acetyl-S-(benzyl)-L-cysteine	BZMA	Toluene; Benzyl alcohol
2-Methylhippuric acid	2MHA	*o*-Xylene
3-Methylhippuric acid + 4-Methylhippuric acid	34MH	*m,p*-Xylene

**Table 3:** Summary of sample-weighted multivariate regression modeling of predictor variables for six urinary VOC metabolites and selected demographics.

VOCM	Sex ^a^	Age ^b^ (% Increase/Decrease)			Race/Ethnicity ^c^ (% Increase/Decrease)		
		18–24	35–54	≥55	Non-Hispanic Black	Hispanic	Other Race/Multiracial
2COEMA	9.0	−17	12	32	NSA	−15	−3.1
3HPMA	NSA	−17	16	17	−26	NSA	29
3HMPMA	13	−20	17	36	−28	−18	−4.5
4HMBEMA	23	−24	13	33	−28	−25	−11
t4HBEMA	9.8	−18	11	28	−21	−17	NSA
2CYEMA	NSA						

^a^ Reference Group: Males; ^b^ Reference Group: 25–34 year olds; ^c^ Reference Group: Non-Hispanic Whites; ^NSA^ Not statistically significant.

**Table 4:** Weighted geometric means (GMs, ng/mL) comparisons of six selected VOCMs in the PATH Study Wave 1 and the National Health and Nutrition Examination Survey (NHANES) 2015–2016 data stratified by smokers and never users.

Variable	Smokers					Never Users				
	NHANES 2015–2016		PATH Study Wave 1 2013–2014		*p*-Value	NHANES 2015–2016		PATH Study Wave 1 2013–2014		*p*-Value
	*n*	GM (SE)	*n*	GM (SE)		*n*	GM (SE)	*n*	GM (SE)	
t4HBEMA	888	15.7 (1.32)	8080	18.9 (0.444)	0.0208	1331	3.54 (0.15)	3153	4.28 (0.081)	<0.0001
3HPMA	842	725 (39.7)	7973	841 (14.9)	0.0064	1239	221 (7.12)	3153	255 (5.78)	0.0002
2COEMA	888	178 (10.1)	7637	218 (3.17)	0.0001	1331	91.8 (3.03)	3022	92.2 (1.77)	0.9070
2CYEMA	888	58.0 (6.76)	7637	68.4 (2.66)	0.1511	1331	1.41 (0.09)	3022	1.25 (0.034)	0.0967
3HMPMA	888	1.28 × 10^3^ (79.6)	8081	1.62 × 10^3^ (37.1)	0.0001	1331	378 (12.80)	3153	433 (10.2)	0.0009
4HMBEMA	888	18.1 (1.74)	8057	21.4 (0.637)	0.0732	1331	3.22 (0.09)	3120	3.14 (0.088)	0.5241

SE: Standard error; t4HBEMA: *N*-acetyl-S-(4-hydroxy-2-buten-1-yl)-L-cysteine; 3HPMA: *N*-acetyl-S-(3-hydroxypropyl)-L-cysteine; 2COEMA: *N*-acetyl-S-(2-carboxyethyl)-L-cysteine; 2CYEMA: *N*-acetyl-S-(2-cyanoethyl)-L-cysteine; 3HMPMA: *N*-acetyl-S-(3-hydroxypropyl-1-methyl)-L-cysteine; 4HMBEMA: *N*-acetyl-S-(4-hydroxy-2-methyl-2-buten-1-yl)-L-cysteine.

## Supplementary Materials

The following are available online at https://www.mdpi.com/1660-4601/17/15/5408/s1.
