# Supplementary material for: Urinary Biomarkers of Exposure to Volatile Organic Compounds from the Population Assessment of Tobacco and Health Study Wave 1 (2013–2014)
Source: Int J Environ Res Public Health. 2020 Jul 28;17(15):5408. doi: 10.3390/ijerph17155408 (PMC7432690; doi:10.3390/ijerph17155408)
Supplement: Supplementary file 1 [file ijerph-17-05408-s001.pdf]

Table S1. Sample-weighted detection frequencies (percent) for 20 VOCM by tobacco user group.

Analyte	Every Day Established Exclusive Combustible Users [N = 3,156]	Every Day Established Exclusive E-Cigarette Users [N = 149]	Every Day Established Exclusive Smokeless Users [N = 353]	Never Users [N = 1,563]	All [N = 11,501]
2MHA	99.7	90.5	94.0	91.5	94.6
34MH	99.9	99.7	100	99.8	99.8
2CAEMA	100	100	100	99.8	99.8
MCAMA	99.9	100	100	99.8	99.7
BZMA	99.6	100	99.6	99.5	99.5
2COEMA	100	100	99.4	99.5	99.6
1CYHEMA	95.4	15.6	4.67	2.83	36.0
2CYEMA	99.8	93.4	85.4	84.6	91.2
34HBMA	100	100	100	99.8	99.9
2CAHEMA	76.5	42.0	38.0	37.5	51.6
2HEMA	88.5	45.6	47.1	52.7	63.7
2HPMA	99.6	93.5	96.1	97.2	97.7
3HPMA	100	100	100	99.6	99.8
3HMPMA	100	98.8	100	99.9	99.9
4HMBEMA	99.6	81.5	87.9	90.2	93.5
MADA	99.9	96.6	98.6	99.1	99.1
t4HBEMA	99.9	97.9	99.3	98.8	98.9
PHGA	99.9	100	100	99.7	99.7
PHMA	68.5	59.9	65.7	68.3	67.4
TTCA	74.1	62.4	65.7	69.0	70.9

Table S2. Sample-weighted geometric means [standard error] by demographic and tobacco user group for PATH Study Wave 1 (2013-2014): 2-Methylhippuric acid (2MHA).

2MHA		Every Day Established Combustible Users		Every Day Established E-Cigarette Users		Every Day Established Smokeless Users		Never Users	
**Predictor**	**Level**	**Non-Creatinine Ratioed [ng/mL]**	**Creatinine Ratioed [µg/g]**	**Non-Creatinine Ratioed [ng/mL]**	**Creatinine Ratioed [µg/g]**	**Non-Creatinine Ratioed [ng/mL]**	**Creatinine Ratioed [µg/g]**	**Non-Creatinine Ratioed [ng/mL]**	**Creatinine Ratioed [µg/g]**
All	All	113 [2.87]	116 [2.78]	28.1 [2.53]	32.1 [2.42]	35.2 [2.74]	34.2 [2.63]	23.4 [0.906]	22.2 [0.857]
Age	18 – 24	87.6 [7.78]	73.8 [6.10]	27.5 [8.24]†	32.8 [6.98]†	41.4 [9.14]	29.3 [5.12]	23.4 [1.27]	17.9 [0.991]
	25 – 34	123 [4.89]	104 [4.41]	31.1 [5.29]†	29.4 [4.67]†	45.1 [7.00]	36.0 [6.86]	26.3 [2.51]	21.5 [1.57]
	35 – 54	118 [4.49]	122 [4.97]	32.1 [4.10]	34.7 [3.91]	34.7 [4.12]	33.0 [3.57]	25.5 [1.64]	24.5 [1.92]
	≥55	111 [5.78]	141 [6.11]	19.9 [5.22]†	31.7 [7.42]†	29.1 [4.64]	37.1 [5.08]	19.9 [1.68]	22.2 [1.81]
Sex	Male	121 [4.18]	105 [3.18]	34.0 [4.16]	30.0 [3.35]	36.4 [2.88]	35.1 [2.86]	25.0 [1.91]	20.6 [1.46]
	Female	106 [3.81]	129 [4.43]	24.6 [3.34]	33.7 [4.08]	17.0 [7.20]†	20.5 [10.1]†	22.5 [0.980]	23.2 [1.04]
Race	Non-Hispanic White	120 [3.59]	137 [3.79]	28.0 [2.88]	34.2 [2.75]	34.6 [3.02]	34.1 [3.03]	25.3 [1.43]	25.6 [1.43]
	Non-Hispanic Black	111 [7.76]	78.1 [4.56]	53.6 [22.5]†	27.0 [10.2]†	20.8 [4.61]†	15.6 [1.96]†	21.7 [1.89]	16.7 [1.65]
	Hispanic	86.3 [5.86]	75.9 [4.74]	26.6 [7.09]†	26.7 [11.4]†	47.4 [18.3]†	54.8 [17.0]†	22.6 [1.52]	20.3 [1.34]
	Other Race/Multiracial Ethnicity	88.2 [9.44]	96.7 [9.36]	10.7 [6.03]†	11.5 [6.90]†	62.4 [18.2]†	52.1 [13.2]†	17.5 [2.27]	16.4 [2.59]
Education	<High School/GED	115 [4.47]	119 [4.39]	28.7 [8.00]†	29.9 [8.18]†	31.8 [4.64]	33.8 [4.29]	22.0 [1.88]	20.3 [1.63]
	HS Diploma	122 [5.68]	124 [5.77]	34.3 [6.12]†	33.2 [5.35]†	40.9 [6.24]	40.1 [6.50]	28.3 [2.58]	25.1 [2.02]
	<4y College or Associate Degree	109 [4.76]	109 [5.08]	25.4 [4.51]	31.1 [4.06]	36.1 [3.95]	33.8 [3.56]	23.8 [1.24]	21.5 [1.23]
	Bachelor/Advanced	92.7 [6.49]	109 [5.31]	24.8 [2.98]†	34.7 [5.96]†	27.7 [5.17]†	24.3 [3.69]†	20.4 [1.52]	21.4 [1.63]

† Estimate should be interpreted with caution because it has low statistical precision. It is based on a sample size of less than 50, or the coefficient of variation of the estimate is larger than 30%.

Table S3. Sample-weighted geometric means [standard error] by demographic and tobacco user group for PATH Study Wave 1 (2013-2014): 3-Methylhippuric acid + 4-Methylhippuric acid (34MH).

**34MH**		**Every Day Established Combustible Users**		**Every Day Established E-Cigarette Users**		**Every Day Established Smokeless Users**		**Never Users**	
**Predictor**	**Level**	**Non-Creatinine Ratioed [ng/mL]**	**Creatinine Ratioed [µg/g]**	**Non-Creatinine Ratioed [ng/mL]**	**Creatinine Ratioed [µg/g]**	**Non-Creatinine Ratioed [ng/mL]**	**Creatinine Ratioed [µg/g]**	**Non-Creatinine Ratioed [ng/mL]**	**Creatinine Ratioed [µg/g]**
All	All	763 [19.9]	781 [17.9]	172 [15.6]	195 [13.4]	192 [15.7]	189 [12.4]	161 [6.01]	151 [4.48]
Age	18 – 24	582 [52.3]	489 [35.6]	146 [59.4]†	174 [32.3]†	228 [49.5]	161 [26.1]	164 [9.57]	124 [6.44]
	25 – 34	821 [38.0]	694 [31.0]	184 [37.6]†	174 [25.4]†	250 [32.6]	201 [27.6]	169 [17.9]	137 [10.5]
	35 – 54	807 [31.5]	835 [30.1]	179 [27.8]	196 [22.8]	193 [21.9]	183 [18.1]	164 [10.5]	156 [9.92]
	≥55	739 [37.7]	943 [27.4]	153 [32.2]†	229 [28.7]†	155 [22.8]	200 [16.6]	153 [8.33]	172 [7.68]
Sex	Male	827 [26.5]	715 [21.1]	192 [26.3]	166 [17.9]	196 [17.0]	191 [13.1]	172 [9.34]	140 [6.24]
	Female	702 [27.2]	856 [26.3]	159 [20.4]	218 [19.8]	126 [30.4]†	152 [37.9]†	155 [6.88]	159 [5.07]
Race	Non-Hispanic White	812 [26.4]	928 [24.3]	167 [15.8]	202 [14.0]	189 [17.1]	187 [13.9]	178 [8.10]	179 [6.94]
	Non-Hispanic Black	736 [45.5]	514 [26.5]	296 [89.4]†	159 [44.7]†	146 [36.6]†	129 [34.2]†	143 [12.7]	109 [6.99]
	Hispanic	595 [43.3]	524 [26.6]	209 [64.7]†	209 [51.3]†	227 [97.4]†	262 [83.9]†	140 [9.32]	128 [6.33]
	Other Race/Multiracial Ethnicity	553 [62.9]	609 [55.5]	86.6 [49.2]†	93.3 [39.0]†	312 [90.1]†	266 [64.6]†	142 [21.8]	131 [18.9]
Education	<High School/GED	809 [30.6]	835 [30.7]	205 [47.4]†	201 [41.8]†	178 [35.3]	192 [19.6]	154 [11.8]	145 [9.88]
	HS Diploma	802 [38.6]	809 [36.5]	188 [32.7]†	182 [23.2]†	218 [29.0]	212 [28.1]	188 [16.7]	165 [11.1]
	<4y College or Associate Degree	727 [32.3]	728 [30.6]	170 [27.9]	209 [22.0]	196 [19.6]	187 [14.6]	169 [9.43]	152 [7.79]
	Bachelor/Advanced	596 [49.9]	702 [32.0]	127 [18.8]†	177 [23.6]†	157 [26.1]†	138 [19.4]†	139 [8.99]	143 [8.73]

† Estimate should be interpreted with caution because it has low statistical precision. It is based on a sample size of less than 50, or the coefficient of variation of the estimate is larger than 30%.

Table S4. Sample-weighted geometric means [standard error] by demographic and tobacco user group for PATH Study Wave 1 (2013-2014): *N*-acetyl-S-(2-carbamoylethyl)-L-cysteine (2CAEMA).

**2CAEMA**		**Every Day Established Combustible Users**		**Every Day Established E-Cigarette Users**		**Every Day Established Smokeless Users**		**Never Users**	
**Predictor**	**Level**	**Non-Creatinine Ratioed [ng/mL]**	**Creatinine Ratioed [µg/g]**	**Non-Creatinine Ratioed [ng/mL]**	**Creatinine Ratioed [µg/g]**	**Non-Creatinine Ratioed [ng/mL]**	**Creatinine Ratioed [µg/g]**	**Non-Creatinine Ratioed [ng/mL]**	**Creatinine Ratioed [µg/g]**
All	All	149 [3.71]	152 [2.55]	54.5 [4.69]	61.7 [4.18]	48.7 [2.06]	47.9 [1.76]	48.1 [1.68]	45.0 [1.16]
Age	18 – 24	169 [11.6]	143 [6.07]	58.9 [25.7]†	70.4 [11.8]†	62.0 [6.77]	43.8 [3.72]	54.5 [3.36]	41.4 [2.34]
	25 – 34	182 [7.76]	153 [5.55]	75.2 [12.0]†	71.1 [11.5]†	52.4 [5.60]	42.0 [3.97]	54.3 [3.92]	43.6 [3.63]
	35 – 54	155 [5.90]	160 [4.92]	53.9 [6.30]	59.0 [6.14]	52.2 [3.50]	49.7 [2.89]	47.6 [3.09]	45.3 [2.24]
	≥55	114 [4.59]	145 [4.43]	35.7 [6.56]†	53.4 [5.20]†	38.6 [2.82]	49.8 [3.57]	42.4 [2.32]	47.4 [1.77]
Sex	Male	171 [5.04]	148 [3.14]	69.6 [7.50]	60.2 [7.24]	49.3 [2.23]	48.0 [1.82]	53.4 [3.24]	43.1 [2.23]
	Female	129 [4.12]	157 [4.00]	45.9 [5.23]	62.8 [4.96]	38.1 [6.44]†	45.9 [6.55]†	45.1 [1.95]	46.2 [1.27]
Race	Non-Hispanic White	142 [4.27]	162 [3.17]	53.3 [5.11]	64.3 [4.69]	47.7 [2.16]	47.0 [1.61]	47.0 [2.08]	46.9 [1.45]
	Non-Hispanic Black	192 [9.91]	134 [7.01]	95.6 [21.1]†	51.4 [9.09]†	49.8 [13.3]†	44.1 [8.41]†	47.6 [4.59]	36.3 [1.68]
	Hispanic	145 [7.64]	127 [4.73]	40.6 [17.6]†	40.7 [14.6]†	85.4 [44.3]†	106 [51.0]†	50.0 [4.23]	45.5 [2.79]
	Other Race/Multiracial Ethnicity	126 [10.9]	139 [8.23]	51.7 [18.7]†	55.7 [16.6]†	53.4 [5.15]†	45.6 [6.33]†	51.0 [4.97]	46.8 [4.03]
Education	<High School/GED	152 [5.66]	157 [4.46]	64.4 [12.8]†	63.2 [11.0]†	46.1 [4.17]	50.1 [3.35]	49.1 [4.13]	46.3 [3.08]
	HS Diploma	155 [7.30]	157 [5.67]	59.4 [9.78]†	57.5 [8.89]†	48.1 [4.44]	46.8 [3.56]	50.3 [3.78]	43.6 [2.45]
	<4y College or Associate Degree	148 [5.34]	149 [4.14]	52.2 [8.58]	64.2 [6.44]	50.4 [2.89]	48.2 [2.58]	49.9 [2.61]	44.6 [1.46]
	Bachelor/Advanced	113 [8.25]	131 [5.50]	44.2 [10.2]†	61.8 [10.9]†	51.9 [6.83]†	45.5 [4.30]†	44.3 [3.19]	45.8 [2.30]

† Estimate should be interpreted with caution because it has low statistical precision. It is based on a sample size of less than 50, or the coefficient of variation of the estimate is larger than 30%.

Table S5. Sample-weighted geometric means [standard error] by demographic and tobacco user group for PATH Study Wave 1 (2013-2014): *N*-acetyl-S-(N-methylcarbamoyl)-L-cysteine (MCAMA).

MCAMA		Every Day Established Combustible Users		Every Day Established E-Cigarette Users		Every Day Established Smokeless Users		Never Users	
**Predictor**	**Level**	**Non-Creatinine Ratioed [ng/mL]**	**Creatinine Ratioed [µg/g]**	**Non-Creatinine Ratioed [ng/mL]**	**Creatinine Ratioed [µg/g]**	**Non-Creatinine Ratioed [ng/mL]**	**Creatinine Ratioed [µg/g]**	**Non-Creatinine Ratioed [ng/mL]**	**Creatinine Ratioed [µg/g]**
All	All	527 [10.9]	540 [11.0]	180 [12.5]	203 [12.0]	135 [5.39]	133 [4.77]	111 [3.45]	104 [3.08]
Age	18 – 24	363 [16.0]	304 [10.6]	98.5 [20.9]†	118 [30.8]†	137 [13.9]	97.0 [9.27]	90.0 [5.12]	68.5 [3.45]
	25 – 34	516 [21.5]	438 [19.2]	165 [25.6]†	155 [16.1]†	146 [17.6]	119 [9.99]	104 [7.11]	84.4 [5.02]
	35 – 54	574 [20.8]	594 [21.3]	208 [27.0]	227 [21.2]	146 [8.81]	139 [5.78]	115 [8.28]	109 [6.12]
	≥55	548 [22.0]	700 [24.4]	184 [33.1]†	275 [33.4]†	112 [8.57]	145 [13.2]	121 [6.54]	136 [8.11]
Sex	Male	519 [13.0]	449 [10.3]	187 [24.6]	162 [16.9]	135 [5.86]	132 [5.04]	107 [4.86]	87.0 [4.21]
	Female	535 [17.3]	653 [17.8]	174 [19.0]	238 [19.3]	137 [15.0]†	164 [20.9]†	112 [4.82]	115 [3.71]
Race	Non-Hispanic White	561 [13.7]	642 [14.2]	179 [13.9]	216 [14.2]	134 [6.49]	133 [4.86]	123 [5.24]	123 [5.43]
	Non-Hispanic Black	479 [23.8]	334 [13.9]	242 [29.4]†	130 [17.3]†	129 [26.2]†	114 [21.6]†	82.8 [7.77]	63.2 [3.57]
	Hispanic	446 [30.6]	391 [17.1]	169 [30.1]†	170 [30.7]†	168 [54.6]†	194 [60.6]†	106 [5.71]	96.8 [5.01]
	Other Race/Multiracial Ethnicity	396 [39.2]	438 [34.1]	115 [41.9]†	124 [42.0]†	145 [16.7]†	124 [18.5]†	95.7 [10.7]	88.0 [8.71]
Education	<High School/GED	532 [19.7]	550 [21.5]	200 [36.7]†	197 [23.0]†	135 [8.52]	146 [13.1]	117 [8.52]	110 [7.14]
	HS Diploma	562 [20.7]	567 [22.1]	203 [33.3]†	197 [20.6]†	138 [12.7]	135 [7.69]	118 [6.42]	103 [6.00]
	<4y College or Associate Degree	514 [16.9]	514 [17.3]	161 [19.1]	198 [20.9]	133 [8.23]	127 [7.23]	107 [7.02]	96.0 [5.16]
	Bachelor/Advanced	422 [32.2]	500 [24.9]	170 [22.3]†	238 [20.7]†	135 [19.6]†	119 [11.9]†	105 [7.06]	109 [5.70]

† Estimate should be interpreted with caution because it has low statistical precision. It is based on a sample size of less than 50, or the coefficient of variation of the estimate is larger than 30%.

Table S6. Sample-weighted geometric means [standard error] by demographic and tobacco user group for PATH Study Wave 1 (2013-2014): *N*-acetyl-S-(benzyl)-L-cysteine (BZMA).

BZMA		Every Day Established Combustible Users		Every Day Established E-Cigarette Users		Every Day Established Smokeless Users		Never Users	
**Predictor**	**Level**	**Non-Creatinine Ratioed [ng/mL]**	**Creatinine Ratioed [µg/g]**	**Non-Creatinine Ratioed [ng/mL]**	**Creatinine Ratioed [µg/g]**	**Non-Creatinine Ratioed [ng/mL]**	**Creatinine Ratioed [µg/g]**	**Non-Creatinine Ratioed [ng/mL]**	**Creatinine Ratioed [µg/g]**
All	All	6.42 [0.199]	6.58 [0.179]	6.58 [0.590]	7.45 [0.659]	5.55 [0.235]	5.47 [0.198]	6.50 [0.242]	6.10 [0.189]
Age	18 – 24	6.27 [0.379]	5.28 [0.188]	4.41 [1.36]†	5.28 [0.780]†	5.26 [0.689]	3.72 [0.314]	5.90 [0.314]	4.49 [0.184]
	25 – 34	6.98 [0.295]	5.92 [0.205]	7.11 [1.25]†	6.72 [0.960]†	6.30 [0.856]	5.11 [0.630]	7.23 [0.585]	5.86 [0.322]
	35 – 54	6.99 [0.335]	7.22 [0.362]	7.98 [1.32]	8.73 [1.28]	5.81 [0.420]	5.54 [0.319]	6.70 [0.495]	6.37 [0.331]
	≥55	5.34 [0.395]	6.83 [0.385]	4.79 [0.732]†	7.16 [1.41]†	4.83 [0.518]	6.27 [0.468]	6.22 [0.452]	6.97 [0.476]
Sex	Male	7.01 [0.280]	6.07 [0.264]	6.15 [0.955]	5.33 [0.717]	5.56 [0.247]	5.43 [0.206]	6.20 [0.402]	5.02 [0.293]
	Female	5.87 [0.261]	7.16 [0.252]	6.90 [0.846]	9.45 [1.04]	5.39 [1.08]†	6.49 [0.797]†	6.69 [0.339]	6.87 [0.260]
Race	Non-Hispanic White	5.60 [0.195]	6.40 [0.210]	6.28 [0.617]	7.59 [0.771]	5.65 [0.265]	5.59 [0.235]	5.97 [0.312]	5.98 [0.287]
	Non-Hispanic Black	10.8 [0.804]	7.56 [0.555]	11.0 [2.11]†	5.90 [0.647]†	7.81 [2.32]†	6.92 [1.26]†	9.91 [0.789]	7.57 [0.522]
	Hispanic	6.95 [0.433]	6.13 [0.400]	8.41 [3.73]†	8.43 [2.31]†	3.10 [0.484]†	3.58 [0.378]†	6.01 [0.515]	5.48 [0.349]
	Other Race/Multiracial Ethnicity	6.35 [0.546]	6.99 [0.482]	5.58 [1.59]†	6.01 [1.45]†	4.13 [0.622]†	3.67 [0.427]†	7.04 [1.14]	6.47 [0.833]
Education	<High School/GED	6.25 [0.426]	6.47 [0.372]	6.69 [1.87]†	6.56 [1.64]†	5.02 [0.551]	5.43 [0.468]	6.36 [0.476]	5.97 [0.368]
	HS Diploma	6.24 [0.439]	6.31 [0.402]	6.01 [1.08]†	5.82 [1.02]†	5.52 [0.413]	5.40 [0.423]	5.88 [0.383]	5.15 [0.287]
	<4y College or Associate Degree	6.66 [0.224]	6.66 [0.176]	6.69 [0.896]	8.23 [1.16]	5.43 [0.500]	5.22 [0.427]	6.42 [0.379]	5.77 [0.292]
	Bachelor/Advanced	6.94 [0.681]	8.17 [0.829]	7.33 [1.68]†	10.3 [1.88]†	7.33 [1.09]†	6.44 [0.908]†	7.23 [0.544]	7.48 [0.405]

† Estimate should be interpreted with caution because it has low statistical precision. It is based on a sample size of less than 50, or the coefficient of variation of the estimate is larger than 30%.

Table S7. Sample-weighted geometric means [standard error] by demographic and tobacco user group for PATH Study Wave 1 (2013-2014): *N*-acetyl-S-(2-carboxyethyl)-L-cysteine (2COEMA).

2COEMA		Every Day Established Combustible Users		Every Day Established E-Cigarette Users		Every Day Established Smokeless Users		Never Users	
**Predictor**	**Level**	**Non-Creatinine Ratioed [ng/mL]**	**Creatinine Ratioed [µg/g]**	**Non-Creatinine Ratioed [ng/mL]**	**Creatinine Ratioed [µg/g]**	**Non-Creatinine Ratioed [ng/mL]**	**Creatinine Ratioed [µg/g]**	**Non-Creatinine Ratioed [ng/mL]**	**Creatinine Ratioed [µg/g]**
All	All	293 [7.77]	299 [6.31]	102 [8.17]	115 [7.01]	96.8 [5.98]	95.8 [4.00]	100 [3.27]	93.7 [2.26]
Age	18 – 24	208 [17.5]	176 [9.49]	66.3 [20.7]†	80.8 [14.5]†	89.3 [11.3]	64.6 [5.43]	97.7 [4.30]	74.1 [2.07]
	25 – 34	292 [15.6]	246 [11.2]	123 [13.6]†	112 [13.7]†	96.2 [12.3]	75.9 [7.35]	107 [7.66]	85.7 [5.67]
	35 – 54	308 [13.5]	319 [10.8]	93.0 [12.6]	105 [8.36]	97.7 [6.18]	94.1 [5.32]	98.9 [5.91]	93.6 [4.09]
	≥55	316 [17.8]	399 [13.4]	103 [26.8]†	149 [16.6]†	98.0 [13.2]	126 [9.70]	99.2 [6.03]	112 [5.25]
Sex	Male	313 [11.1]	271 [6.06]	131 [12.6]	115 [11.8]	96.2 [6.15]	94.2 [3.96]	110 [5.48]	88.2 [3.37]
	Female	274 [11.3]	331 [12.4]	84.6 [9.31]	115 [6.85]	111 [33.6]†	133 [33.0]†	95.0 [4.17]	97.1 [3.24]
Race	Non-Hispanic White	283 [9.29]	322 [8.22]	92.7 [8.57]	111 [7.51]	93.4 [6.84]	92.9 [4.25]	95.6 [4.03]	95.5 [2.82]
	Non-Hispanic Black	385 [18.3]	270 [12.2]	253 [32.1]†	136 [13.3]†	174 [47.6]†	154 [19.0]†	131 [12.7]	99.6 [4.92]
	Hispanic	263 [14.4]	228 [9.08]	109 [30.7]†	122 [20.7]†	108 [44.1]†	134 [43.8]†	94.5 [6.75]	86.4 [5.21]
	Other Race/Multiracial Ethnicity	222 [21.9]	245 [18.1]	148 [51.6]†	159 [50.5]†	109 [12.5]†	93.2 [10.8]†	102 [8.78]	91.0 [5.36]
Education	<High School/GED	303 [13.6]	305 [11.7]	112 [21.1]†	108 [15.1]†	112 [13.5]	121 [9.59]	110 [8.49]	103 [6.68]
	HS Diploma	311 [13.9]	315 [9.27]	125 [18.2]†	118 [16.0]†	87.3 [7.42]	86.4 [5.67]	104 [6.67]	90.2 [5.54]
	<4y College or Associate Degree	278 [12.1]	279 [13.1]	86.7 [15.5]	110 [9.35]	94.1 [9.54]	90.3 [7.11]	100 [6.69]	89.9 [3.23]
	Bachelor/Advanced	254 [19.3]	300 [18.6]	94.5 [18.5]†	126 [14.1]†	100 [13.1]†	86.9 [10.1]†	92.7 [5.16]	95.3 [3.82]

† Estimate should be interpreted with caution because it has low statistical precision. It is based on a sample size of less than 50, or the coefficient of variation of the estimate is larger than 30%.

Table S8. Sample-weighted geometric means [standard error] by demographic and tobacco user group for PATH Study Wave 1 (2013-2014): *N*-acetyl-S-(1-cyano-2-hydroxyethyl)-L-cysteine (1CYHEMA).

1CY2HEMA		Every Day Established Combustible Users		Every Day Established E-Cigarette Users		Every Day Established Smokeless Users		Never Users	
**Predictor**	**Level**	**Non-Creatinine Ratioed [ng/mL]**	**Creatinine Ratioed [µg/g]**	**Non-Creatinine Ratioed [ng/mL]**	**Creatinine Ratioed [µg/g]**	**Non-Creatinine Ratioed [ng/mL]**	**Creatinine Ratioed [µg/g]**	**Non-Creatinine Ratioed [ng/mL]**	**Creatinine Ratioed [µg/g]**
All	All	27.5 [0.822]	28.2 [0.734]	2.43 [0.164]	2.75 [0.227]	2.00 [0.035]	1.96 [0.094]	1.94 [0.023]	1.82 [0.057]
Age	18 – 24	21.1 [1.90]	17.8 [1.18]	3.52 [0.871]	4.21 [1.30]	1.98 [0.089]	1.40 [0.177]	2.07 [0.118]	1.57 [0.106]
	25 – 34	29.2 [1.89]	24.7 [1.44]	3.06 [0.581]	2.90 [0.608]	2.17 [0.149]	1.75 [0.189]	2.00 [0.080]	1.63 [0.099]
	35 – 54	29.8 [1.41]	30.9 [1.30]	2.13 [0.130]	2.33 [0.261]	2.03 [0.096]	1.93 [0.123]	1.87 [0.014]	1.77 [0.105]
	≥55	26.0 [1.65]	33.1 [1.86]	2.03 [0.141]	3.03 [0.519]	1.85 [0.010]	2.39 [0.246]	1.93 [0.037]	2.16 [0.102]
Sex	Male	28.6 [1.22]	24.7 [0.852]	2.62 [0.326]	2.27 [0.350]	2.01 [0.037]	1.95 [0.104]	2.02 [0.058]	1.64 [0.070]
	Female	26.5 [1.12]	32.3 [1.32]	2.30 [0.181]	3.15 [0.297]	1.84 [0.00]	2.21 [0.367]	1.89 [0.016]	1.94 [0.078]
Race	Non-Hispanic White	29.0 [0.916]	33.1 [0.898]	2.35 [0.152]	2.84 [0.262]	1.96 [0.032]	1.93 [0.102]	1.94 [0.033]	1.94 [0.088]
	Non-Hispanic Black	28.0 [2.12]	19.5 [1.41]	3.66 [1.67]	1.97 [0.968]	2.06 [0.275]	1.83 [0.341]	1.97 [0.052]	1.50 [0.105]
	Hispanic	22.1 [2.52]	19.5 [1.63]	2.67 [1.13]	2.68 [1.07]	3.58 [2.50]	4.14 [2.97]	1.88 [0.014]	1.72 [0.088]
	Other Race/Multiracial Ethnicity	18.8 [2.29]	20.7 [2.15]	2.21 [0.539]	2.38 [0.923]	1.92 [0.074]	1.64 [0.270]	2.02 [0.147]	1.86 [0.208]
Education	<High School/GED	29.9 [1.53]	30.9 [1.54]	2.25 [0.310]	2.21 [0.395]	2.01 [0.095]	2.18 [0.282]	2.01 [0.113]	1.89 [0.135]
	HS Diploma	29.7 [1.61]	30.0 [1.53]	2.60 [0.393]	2.52 [0.537]	2.09 [0.139]	2.03 [0.187]	1.94 [0.036]	1.70 [0.098]
	<4y College or Associate Degree	25.2 [1.30]	25.2 [1.33]	2.48 [0.254]	3.05 [0.351]	1.94 [0.052]	1.86 [0.124]	1.93 [0.040]	1.74 [0.078]
	Bachelor/Advanced	20.9 [1.71]	24.7 [1.91]	2.18 [0.252]	3.05 [0.466]	1.88 [0.038]	1.65 [0.167]	1.91 [0.042]	1.97 [0.110]

† Estimate should be interpreted with caution because it has low statistical precision. It is based on a sample size of less than 50, or the coefficient of variation of the estimate is larger than 30%.

Table S9. Sample-weighted geometric means [standard error] by demographic and tobacco user group for PATH Study Wave 1 (2013-2014): *N*-acetyl-S-(2-cyanoethyl)-L-cysteine (2CYEMA).

2CYEMA		Every Day Established Combustible Users		Every Day Established E-Cigarette Users		Every Day Established Smokeless Users		Never Users	
**Predictor**	**Level**	**Non-Creatinine Ratioed [ng/mL]**	**Creatinine Ratioed [µg/g]**	**Non-Creatinine Ratioed [ng/mL]**	**Creatinine Ratioed [µg/g]**	**Non-Creatinine Ratioed [ng/mL]**	**Creatinine Ratioed [µg/g]**	**Non-Creatinine Ratioed [ng/mL]**	**Creatinine Ratioed [µg/g]**
All	All	168 [4.92]	172 [4.77]	3.99 [0.549]	4.51 [0.560]	1.79 [0.151]	1.76 [0.114]	1.35 [0.049]	1.27 [0.043]
Age	18 – 24	106 [10.5]	89.4 [6.80]	5.65 [3.28]†	6.75 [3.02]†	2.85 [0.640]	2.01 [0.393]	1.53 [0.154]	1.17 [0.117]
	25 – 34	181 [9.75]	153 [7.97]	6.14 [2.20]†	5.80 [2.06]†	2.45 [0.412]	1.97 [0.337]	1.51 [0.141]	1.22 [0.097]
	35 – 54	191 [7.80]	198 [8.21]	3.89 [0.882]	4.25 [0.860]	1.80 [0.168]	1.72 [0.171]	1.30 [0.091]	1.23 [0.081]
	≥55	158 [8.12]	202 [8.87]	2.17 [0.681]†	3.24 [0.689]†	1.27 [0.220]	1.64 [0.189]	1.24 [0.079]	1.39 [0.081]
Sex	Male	186 [6.48]	161 [5.09]	5.09 [1.24]	4.40 [1.07]	1.80 [0.163]	1.75 [0.123]	1.52 [0.107]	1.23 [0.076]
	Female	152 [6.24]	185 [7.47]	3.36 [0.579]	4.60 [0.623]	1.66 [0.389]†	1.99 [0.552]†	1.26 [0.057]	1.29 [0.053]
Race	Non-Hispanic White	169 [5.25]	193 [6.19]	3.86 [0.583]	4.66 [0.655]	1.72 [0.156]	1.70 [0.119]	1.24 [0.053]	1.24 [0.057]
	Non-Hispanic Black	213 [14.2]	149 [10.1]	12.3 [7.16]†	6.62 [3.75]†	2.07 [0.874]†	1.83 [0.544]†	1.81 [0.253]	1.39 [0.156]
	Hispanic	123 [12.5]	108 [9.67]	2.09 [1.55]†	2.09 [1.29]†	2.99 [3.43]†	3.45 [4.10]†	1.37 [0.115]	1.25 [0.086]
	Other Race/Multiracial Ethnicity	125 [18.9]	138 [17.3]	2.66 [2.09]†	2.86 [2.20]†	2.66 [0.513]†	2.27 [0.456]†	1.43 [0.196]	1.32 [0.147]
Education	<High School/GED	177 [8.19]	183 [8.44]	5.41 [2.38]†	5.31 [1.93]†	1.91 [0.349]	2.07 [0.265]	1.53 [0.203]	1.44 [0.182]
	HS Diploma	184 [9.37]	185 [8.76]	4.93 [1.43]†	4.78 [1.45]†	1.74 [0.242]	1.69 [0.241]	1.49 [0.141]	1.31 [0.107]
	<4y College or Associate Degree	156 [7.88]	156 [7.30]	3.82 [1.01]	4.69 [0.968]	1.79 [0.206]	1.71 [0.183]	1.32 [0.091]	1.19 [0.069]
	Bachelor/Advanced	133 [11.7]	157 [12.4]	2.24 [0.575]†	3.13 [0.828]†	1.69 [0.363]†	1.49 [0.209]†	1.18 [0.074]	1.22 [0.064]

† Estimate should be interpreted with caution because it has low statistical precision. It is based on a sample size of less than 50, or the coefficient of variation of the estimate is larger than 30%.

Table S10. Sample-weighted geometric means [standard error] by demographic and tobacco user group for PATH Study Wave 1 (2013-2014): *N*-acetyl-S-(3,4-dihydroxybutyl)-L-cysteine (34HBMA).

34HBMA		Every Day Established Combustible Users		Every Day Established E-Cigarette Users		Every Day Established Smokeless Users		Never Users	
**Predictor**	**Level**	**Non-Creatinine Ratioed [ng/mL]**	**Creatinine Ratioed [µg/g]**	**Non-Creatinine Ratioed [ng/mL]**	**Creatinine Ratioed [µg/g]**	**Non-Creatinine Ratioed [ng/mL]**	**Creatinine Ratioed [µg/g]**	**Non-Creatinine Ratioed [ng/mL]**	**Creatinine Ratioed [µg/g]**
All	All	501 [8.84]	516 [6.27]	335 [20.5]	386 [11.5]	369 [13.1]	361 [8.26]	370 [11.1]	347 [6.17]
Age	18 – 24	474 [25.7]	394 [6.83]	265 [92.3]†	324 [15.9]†	402 [37.8]	277 [12.6]	383 [13.6]	295 [6.81]
	25 – 34	533 [20.1]	452 [14.9]	364 [37.6]†	354 [21.9]†	427 [42.1]	318 [21.4]	375 [25.7]	293 [17.4]
	35 – 54	513 [16.9]	532 [11.4]	341 [34.4]	374 [14.3]	364 [17.0]	356 [11.2]	362 [20.5]	348 [9.36]
	≥55	470 [19.1]	616 [12.0]	308 [55.9]†	480 [36.4]†	338 [28.1]	431 [20.4]	370 [16.5]	415 [11.9]
Sex	Male	561 [12.0]	484 [7.91]	383 [33.3]	349 [16.1]	370 [13.9]	359 [8.92]	387 [16.4]	317 [11.3]
	Female	444 [12.7]	553 [10.0]	306 [25.1]	414 [17.8]	338 [46.3]†	417 [51.8]†	361 [13.8]	366 [6.21]
Race	Non-Hispanic White	486 [11.0]	556 [7.67]	325 [24.3]	401 [14.3]	369 [14.9]	361 [9.02]	379 [16.1]	381 [8.66]
	Non-Hispanic Black	572 [18.6]	406 [12.8]	567 [130]†	311 [39.4]†	398 [57.5]†	371 [29.3]†	394 [18.9]	283 [9.22]
	Hispanic	531 [27.0]	472 [13.3]	317 [69.7]†	318 [42.7]†	345 [63.3]†	428 [99.2]†	354 [22.1]	323 [12.7]
	Other Race/Multiracial Ethnicity	433 [31.5]	488 [23.1]	295 [50.6]†	318 [47.9]†	357 [44.2]†	323 [30.0]†	319 [23.9]	298 [14.2]
Education	<High School/GED	511 [14.7]	533 [10.3]	413 [62.4]†	440 [49.0]†	369 [28.5]	396 [30.8]	388 [17.2]	357 [11.6]
	HS Diploma	510 [16.6]	523 [13.5]	353 [43.6]†	349 [19.9]†	366 [22.4]	358 [15.1]	369 [26.3]	330 [17.4]
	<4y College or Associate Degree	499 [13.3]	496 [10.4]	316 [37.3]	390 [20.5]	362 [21.9]	343 [10.9]	379 [18.7]	341 [9.66]
	Bachelor/Advanced	434 [34.5]	512 [15.1]	290 [51.3]†	400 [21.3]†	397 [40.8]†	349 [24.2]†	355 [20.0]	360 [12.3]

† Estimate should be interpreted with caution because it has low statistical precision. It is based on a sample size of less than 50, or the coefficient of variation of the estimate is larger than 30%.

Table S11. Sample-weighted geometric means [standard error] by demographic and tobacco user group for PATH Study Wave 1 (2013-2014): *N*-acetyl-S-(2-carbamoyl-2-hydroxyethyl)-L-cysteine (2CAHEMA).

2CA2HEMA		Every Day Established Combustible Users		Every Day Established E-Cigarette Users		Every Day Established Smokeless Users		Never Users	
**Predictor**	**Level**	**Non-Creatinine Ratioed [ng/mL]**	**Creatinine Ratioed [µg/g]**	**Non-Creatinine Ratioed [ng/mL]**	**Creatinine Ratioed [µg/g]**	**Non-Creatinine Ratioed [ng/mL]**	**Creatinine Ratioed [µg/g]**	**Non-Creatinine Ratioed [ng/mL]**	**Creatinine Ratioed [µg/g]**
All	All	18.4 [0.362]	18.8 [0.255]	10.1 [0.528]	11.4 [0.743]	8.95 [0.217]	8.72 [0.333]	9.11 [0.149]	8.57 [0.253]
Age	18 – 24	21.3 [1.24]	18.0 [0.612]	10.8 [2.18]	12.9 [2.98]	10.5 [1.07]	7.43 [0.835]	9.57 [0.299]	7.31 [0.228]
	25 – 34	22.3 [0.958]	18.7 [0.696]	11.3 [1.33]	10.8 [1.53]	9.41 [0.592]	7.46 [0.535]	9.97 [0.562]	8.14 [0.606]
	35 – 54	18.3 [0.634]	19.0 [0.465]	10.2 [0.923]	11.3 [1.37]	9.11 [0.384]	8.75 [0.467]	8.99 [0.238]	8.60 [0.458]
	≥55	15.0 [0.535]	19.0 [0.548]	8.45 [0.618]	12.0 [1.51]	8.02 [0.319]	10.0 [0.719]	8.58 [0.261]	9.52 [0.445]
Sex	Male	20.1 [0.523]	17.3 [0.364]	10.7 [0.905]	9.33 [1.05]	9.01 [0.223]	8.68 [0.365]	9.08 [0.313]	7.33 [0.338]
	Female	16.9 [0.481]	20.5 [0.397]	9.66 [0.716]	13.3 [1.09]	7.76 [0.641]	9.46 [1.27]	9.13 [0.220]	9.41 [0.354]
Race	Non-Hispanic White	18.0 [0.458]	20.5 [0.360]	9.99 [0.552]	12.0 [0.876]	8.86 [0.223]	8.66 [0.347]	9.11 [0.173]	9.12 [0.391]
	Non-Hispanic Black	20.9 [0.848]	14.6 [0.515]	13.9 [3.46]	7.64 [1.62]	8.84 [1.33]	7.82 [0.944]	8.91 [0.384]	6.88 [0.416]
	Hispanic	18.4 [1.04]	16.4 [0.672]	8.92 [2.86]	8.93 [2.84]	12.7 [4.56]	15.5 [5.53]	9.52 [0.380]	8.71 [0.368]
	Other Race/Multiracial Ethnicity	16.6 [1.10]	18.6 [1.07]	9.05 [2.29]	9.75 [3.32]	8.78 [0.516]	7.44 [0.959]	8.47 [0.560]	7.79 [0.775]
Education	<High School/GED	18.9 [0.667]	19.4 [0.476]	10.5 [1.81]	10.4 [1.70]	8.87 [0.483]	9.42 [0.975]	9.00 [0.357]	8.48 [0.451]
	HS Diploma	18.9 [0.783]	19.1 [0.600]	10.9 [1.20]	10.5 [1.56]	9.13 [0.499]	8.91 [0.605]	9.46 [0.394]	8.21 [0.480]
	<4y College or Associate Degree	18.5 [0.499]	18.3 [0.484]	9.57 [0.743]	11.6 [1.10]	8.85 [0.319]	8.41 [0.455]	9.39 [0.252]	8.48 [0.358]
	Bachelor/Advanced	14.7 [0.952]	17.4 [0.817]	9.75 [1.50]	14.0 [2.24]	8.87 [0.772]	7.68 [0.692]	8.64 [0.262]	9.00 [0.431]

† Estimate should be interpreted with caution because it has low statistical precision. It is based on a sample size of less than 50, or the coefficient of variation of the estimate is larger than 30%.

Table S12. Sample-weighted geometric means [standard error] by demographic and tobacco user group for PATH Study Wave 1 (2013-2014): *N*-acetyl-S-(2-hydroxyethyl)-L-cysteine (2HEMA).

2HEMA		Every Day Established Combustible Users		Every Day Established E-Cigarette Users		Every Day Established Smokeless Users		Never Users	
**Predictor**	**Level**	**Non-Creatinine Ratioed [ng/mL]**	**Creatinine Ratioed [µg/g]**	**Non-Creatinine Ratioed [ng/mL]**	**Creatinine Ratioed [µg/g]**	**Non-Creatinine Ratioed [ng/mL]**	**Creatinine Ratioed [µg/g]**	**Non-Creatinine Ratioed [ng/mL]**	**Creatinine Ratioed [µg/g]**
All	All	3.04 [0.09117]	3.11 [0.084]	0.928 [0.052]	1.09 [0.076]	0.898 [0.027]	0.878 [0.040]	1.04 [0.035]	0.965 [0.037]
Age	18 – 24	2.87 [0.298]	2.41 [0.170]	1.06 [0.296]†	1.30 [0.500]†	0.920 [0.070]	0.643 [0.079]	1.13 [0.065]	0.833 [0.045]
	25 – 34	3.51 [0.226]	2.95 [0.150]	0.985 [0.115]†	0.941 [0.166]†	0.944 [0.082]	0.701 [0.075]	1.11 [0.088]	0.911 [0.074]
	35 – 54	3.46 [0.173]	3.55 [0.188]	0.992 [0.102]	1.11 [0.113]	0.908 [0.046]	0.878 [0.050]	1.08 [0.055]	1.01 [0.062]
	≥55	2.32 [0.122]	2.99 [0.141]	0.749 [0.061]†	1.21 [0.168]†	0.852 [0.046]	1.09 [0.105]	0.920 [0.069]	1.01 [0.082]
Sex	Male	2.84 [0.105]	2.45 [0.080]	0.807 [0.049]	0.716 [0.068]	0.902 [0.027]	0.873 [0.041]	0.918 [0.053]	0.738 [0.045]
	Female	3.26 [0.147]	3.98 [0.158]	1.02 [0.092]	1.46 [0.106]	0.805 [0.107]†	0.991 [0.174]†	1.12 [0.048]	1.13 [0.055]
Race	Non-Hispanic White	3.01 [0.110]	3.45 [0.109]	0.883 [0.052]	1.10 [0.086]	0.887 [0.029]	0.872 [0.042]	0.970 [0.052]	0.951 [0.054]
	Non-Hispanic Black	3.38 [0.257]	2.36 [0.184]	1.82 [0.699]†	0.989 [0.300]†	0.984 [0.240]†	0.907 [0.217]†	1.22 [0.088]	0.930 [0.052]
	Hispanic	3.06 [0.218]	2.67 [0.170]	0.808 [0.282]†	0.923 [0.278]†	0.993 [0.234]†	1.15 [0.337]†	1.13 [0.065]	1.03 [0.061]
	Other Race/Multiracial Ethnicity	2.44 [0.305]	2.68 [0.284]	1.04 [0.212]†	1.23 [0.290]†	0.984 [0.151]†	0.802 [0.089]†	1.03 [0.111]	0.950 [0.117]
Education	<High School/GED	3.43 [0.171]	3.50 [0.156]	1.01 [0.118]†	0.989 [0.155]†	0.958 [0.055]	1.05 [0.131]	1.04 [0.064]	0.972 [0.074]
	HS Diploma	3.15 [0.202]	3.13 [0.175]	0.997 [0.122]†	1.03 [0.189]†	0.905 [0.064]	0.881 [0.072]	0.969 [0.054]	0.840 [0.045]
	<4y College or Associate Degree	2.81 [0.136]	2.86 [0.125]	0.933 [0.100]	1.21 [0.121]	0.900 [0.064]	0.831 [0.050]	1.11 [0.060]	0.979 [0.060]
	Bachelor/Advanced	2.26 [0.252]	2.70 [0.321]	0.735 [0.081]†	1.02 [0.141]†	0.765 [0.059]†	0.682 [0.064]†	1.04 [0.078]	1.07 [0.072]

† Estimate should be interpreted with caution because it has low statistical precision. It is based on a sample size of less than 50, or the coefficient of variation of the estimate is larger than 30%.

Table S13. Sample-weighted geometric means [standard error] by demographic and tobacco user group for PATH Study Wave 1 (2013-2014): *N*-acetyl-S-(2-hydroxypropyl)-L-cysteine (2HPMA).

2HPMA		Every Day Established Combustible Users		Every Day Established E-Cigarette Users		Every Day Established Smokeless Users		Never Users	
**Predictor**	**Level**	**Non-Creatinine Ratioed [ng/mL]**	**Creatinine Ratioed [µg/g]**	**Non-Creatinine Ratioed [ng/mL]**	**Creatinine Ratioed [µg/g]**	**Non-Creatinine Ratioed [ng/mL]**	**Creatinine Ratioed [µg/g]**	**Non-Creatinine Ratioed [ng/mL]**	**Creatinine Ratioed [µg/g]**
All	All	77.8 [2.06]	79.8 [1.62]	34.7 [3.03]	39.0 [2.71]	28.4 [1.79]	27.8 [1.45]	33.6 [1.67]	31.6 [1.60]
Age	18 – 24	63.9 [6.12]	53.9 [3.56]	23.7 [7.97]†	28.3 [5.09]†	33.2 [4.55]	23.4 [2.29]	34.0 [1.86]	26.0 [1.04]
	25 – 34	87.0 [6.35]	73.4 [3.88]	42.4 [4.98]†	40.0 [5.88]†	32.9 [3.24]	26.4 [2.44]	35.0 [4.30]	28.7 [3.25]
	35 – 54	79.8 [3.22]	82.7 [2.48]	31.9 [4.63]	34.3 [4.14]	27.6 [2.15]	26.2 [2.14]	30.2 [1.64]	28.8 [1.96]
	≥55	74.5 [4.42]	95.3 [4.04]	33.5 [10.7]†	50.0 [9.43]†	25.9 [4.35]	33.5 [4.54]	36.8 [4.70]	41.3 [4.95]
Sex	Male	85.8 [3.14]	74.3 [2.21]	39.7 [6.27]	34.4 [5.00]	28.6 [1.90]	27.7 [1.51]	35.3 [3.57]	28.7 [2.88]
	Female	70.3 [2.90]	85.9 [2.90]	31.5 [4.17]	42.7 [4.13]	25.0 [6.91]†	29.4 [7.38]†	32.5 [1.59]	33.6 [1.67]
Race	Non-Hispanic White	76.4 [2.32]	87.4 [2.14]	32.8 [2.98]	39.3 [2.53]	28.1 [1.96]	27.6 [1.50]	34.4 [2.71]	34.6 [2.75]
	Non-Hispanic Black	86.7 [6.27]	60.6 [4.18]	55.8 [9.99]†	30.0 [6.26]†	29.6 [8.68]†	26.2 [6.69]†	33.3 [2.46]	25.6 [2.76]
	Hispanic	81.3 [7.07]	71.5 [4.36]	32.0 [20.5]†	32.0 [16.6]†	34.5 [12.4]†	39.9 [14.4]†	29.7 [2.39]	27.3 [1.70]
	Other Race/Multiracial Ethnicity	63.6 [7.37]	70.2 [6.37]	69.9 [21.3]†	75.3 [14.9]†	30.6 [6.63]†	26.1 [5.63]†	38.4 [4.93]	35.2 [4.15]
Education	<High School/GED	83.8 [3.78]	86.7 [3.45]	40.7 [8.08]†	40.0 [7.72]†	28.2 [4.75]	30.5 [3.08]	28.9 [1.93]	27.2 [1.57]
	HS Diploma	78.5 [4.06]	79.3 [2.99]	41.5 [7.38]†	40.1 [6.56]†	27.6 [3.28]	26.8 [2.81]	29.7 [2.51]	26.1 [1.79]
	<4y College or Associate Degree	74.9 [3.41]	75.1 [3.45]	29.1 [4.83]	35.8 [3.02]	29.2 [2.60]	27.8 [2.08]	33.5 [1.75]	30.2 [1.78]
	Bachelor/Advanced	65.7 [6.11]	77.3 [4.98]	33.7 [9.28]†	45.6 [9.35]†	28.8 [4.54]†	25.3 [4.26]†	40.2 [5.23]	41.9 [5.68]

† Estimate should be interpreted with caution because it has low statistical precision. It is based on a sample size of less than 50, or the coefficient of variation of the estimate is larger than 30%.

Table S14. Sample-weighted geometric means [standard error] by demographic and tobacco user group for PATH Study Wave 1 (2013-2014): *N*-acetyl-S-(3-hydroxypropyl)-L-cysteine (3HPMA).

HPMA		Every Day Established Combustible Users		Every Day Established E-Cigarette Users		Every Day Established Smokeless Users		Never Users	
**Predictor**	**Level**	**Non-Creatinine Ratioed [ng/mL]**	**Creatinine Ratioed [µg/g]**	**Non-Creatinine Ratioed [ng/mL]**	**Creatinine Ratioed [µg/g]**	**Non-Creatinine Ratioed [ng/mL]**	**Creatinine Ratioed [µg/g]**	**Non-Creatinine Ratioed [ng/mL]**	**Creatinine Ratioed [µg/g]**
All	All	1.29E+03 [31.2]	1.32E+03 [33.0]	309 [26.5]	354 [22.3]	255 [12.8]	249 [10.1]	279 [10.1]	262 [7.66]
Age	18 – 24	797 [90.2]	670 [56.6]	269 [112]†	321 [53.6]†	247 [27.1]	176 [17.2]	286 [13.6]	218 [9.06]
	25 – 34	1.28E+03 [53.7]	1.09E+03 [41.2]	403 [43.2]†	381 [47.6]†	298 [32.0]	244 [20.2]	298 [23.3]	242 [15.4]
	35 – 54	1.44E+03 [52.6]	1.48E+03 [59.7]	284 [33.8]	311 [22.8]	253 [16.8]	239 [14.8]	289 [19.9]	274 [18.1]
	≥55	1.33E+03 [61.8]	1.69E+03 [63.7]	254 [77.0]†	408 [57.6]†	237 [29.6]	302 [24.5]	254 [15.8]	285 [13.8]
Sex	Male	1.37E+03 [34.5]	1.19E+03 [30.5]	383 [34.7]	337 [32.8]	255 [13.2]	247 [9.80]	314 [16.6]	254 [9.12]
	Female	1.21E+03 [52.2]	1.47E+03 [60.5]	267 [33.3]	366 [30.4]	251 [107]†	302 [111]†	260 [12.0]	266 [10.9]
Race	Non-Hispanic White	1.32E+03 [41.4]	1.50E+03 [50.0]	291 [27.9]	357 [23.5]	253 [14.3]	248 [10.2]	267 [11.2]	267 [10.4]
	Non-Hispanic Black	1.37E+03 [67.5]	957 [46.4]	654 [126]†	352 [75.8]†	279 [65.0]†	247 [27.6]†	253 [27.0]	193 [11.5]
	Hispanic	1.07E+03 [62.9]	944 [41.9]	216 [95.2]†	216 [64.8]†	287 [81.2]†	332 [100]†	289 [25.7]	263 [16.4]
	Other Race/Multiracial Ethnicity	1.03E+03 [118]	1.13E+03 [104]	516 [112]†	556 [151]†	267 [49.0]†	228 [50.1]†	403 [46.9]	370 [36.4]
Education	<High School/GED	1.36E+03 [44.9]	1.41E+03 [47.7]	391 [76.0]†	384 [48.9]†	275 [38.1]	299 [24.7]	273 [21.3]	256 [15.2]
	HS Diploma	1.38E+03 [58.9]	1.40E+03 [59.3]	364 [52.4]†	366 [50.5]†	249 [19.3]	237 [15.3]	292 [21.9]	256 [16.7]
	<4y College or Associate Degree	1.19E+03 [63.7]	1.19E+03 [63.0]	268 [49.8]	329 [35.9]	249 [23.2]	240 [17.4]	284 [16.8]	256 [9.91]
	Bachelor/Advanced	1.07E+03 [81.2]	1.25E+03 [72.7]	266 [53.1]†	372 [43.6]†	247 [39.2]†	217 [23.5]†	266 [18.5]	275 [16.4]

† Estimate should be interpreted with caution because it has low statistical precision. It is based on a sample size of less than 50, or the coefficient of variation of the estimate is larger than 30%.

Table S15. Sample-weighted geometric means [standard error] by demographic and tobacco user group for PATH Study Wave 1 (2013-2014): *N*-acetyl-S-(3-hydroxypropyl-1-methyl)-L-cysteine (3HMPMA).

**3HMPMA**		**Every Day Established Combustible Users**		**Every Day Established E-Cigarette Users**		**Every Day Established Smokeless Users**		**Never Users**	
**Predictor**	**Level**	**Non-Creatinine Ratioed [ng/mL]**	**Creatinine Ratioed [µg/g]**	**Non-Creatinine Ratioed [ng/mL]**	**Creatinine Ratioed [µg/g]**	**Non-Creatinine Ratioed [ng/mL]**	**Creatinine Ratioed [µg/g]**	**Non-Creatinine Ratioed [ng/mL]**	**Creatinine Ratioed [µg/g]**
All	All	2.68E+03 [67.9]	2.74E+03 [68.4]	382 [37.2]	432 [36.1]	446 [16.3]	438 [16.0]	471 [19.4]	442 [13.1]
Age	18 – 24	1.63E+03 [153]	1.37E+03 [95.0]	376 [136]†	450 [125]†	403 [41.5]	284 [14.0]	435 [19.0]	331 [11.1]
	25 – 34	2.64E+03 [122]	2.23E+03 [101]	358 [88.8]†	339 [73.2]†	465 [36.3]	374 [29.8]	478 [30.5]	388 [18.9]
	35 – 54	2.98E+03 [110]	3.08E+03 [119]	386 [41.5]	422 [26.1]	454 [23.5]	432 [19.4]	469 [42.6]	445 [27.0]
	≥55	2.82E+03 [143]	3.60E+03 [138]	411 [81.9]†	614 [47.3]†	436 [36.8]	563 [36.1]	491 [34.8]	550 [31.9]
Sex	Male	2.79E+03 [76.9]	2.42E+03 [65.7]	405 [82.6]	350 [59.0]	450 [17.5]	437 [17.1]	499 [27.7]	405 [20.7]
	Female	2.56E+03 [106]	3.12E+03 [116]	367 [32.1]	503 [29.4]	384 [54.3]†	462 [72.7]†	455 [24.8]	467 [15.9]
Race	Non-Hispanic White	2.86E+03 [85.7]	3.26E+03 [94.3]	369 [39.2]	446 [40.9]	446 [17.8]	440 [17.5]	472 [26.0]	472 [18.7]
	Non-Hispanic Black	2.59E+03 [141]	1.81E+03 [103]	701 [127]†	377 [56.3]†	484 [115]†	429 [71.7]†	460 [40.5]	351 [17.0]
	Hispanic	2.00E+03 [118]	1.76E+03 [81.8]	347 [97.0]†	347 [65.4]†	400 [104]†	462 [149]†	466 [45.6]	425 [31.5]
	Other Race/Multiracial Ethnicity	2.02E+03 [247]	2.23E+03 [218]	324 [64.7]†	349 [87.0]†	448 [61.4]†	382 [65.4]†	500 [50.4]	460 [47.8]
Education	<High School/GED	2.79E+03 [95.7]	2.89E+03 [104]	399 [54.1]†	391 [33.2]†	459 [43.0]	497 [41.6]	496 [45.1]	466 [36.8]
	HS Diploma	2.82E+03 [130]	2.85E+03 [139]	472 [77.5]†	457 [53.9]†	465 [25.8]	452 [23.5]	458 [38.4]	401 [25.0]
	<4y College or Associate Degree	2.53E+03 [124]	2.54E+03 [122]	328 [68.8]	403 [70.6]	422 [24.5]	404 [17.0]	463 [22.2]	416 [15.4]
	Bachelor/Advanced	2.26E+03 [172]	2.66E+03 [145]	370 [56.9]†	518 [47.3]†	434 [51.9]†	381 [28.0]†	478 [40.3]	494 [29.4]

† Estimate should be interpreted with caution because it has low statistical precision. It is based on a sample size of less than 50, or the coefficient of variation of the estimate is larger than 30%.

Table S16. Sample-weighted geometric means [standard error] by demographic and tobacco user group for PATH Study Wave 1 (2013-2014): *N*-acetyl-S-(4-hydroxy-2-methyl-2-buten-1-yl)-L-cysteine (4HMBEMA).

4HMBEMA		Every Day Established Combustible Users		Every Day Established E-Cigarette Users		Every Day Established Smokeless Users		Never Users	
**Predictor**	**Level**	**Non-Creatinine Ratioed [ng/mL]**	**Creatinine Ratioed [µg/g]**	**Non-Creatinine Ratioed [ng/mL]**	**Creatinine Ratioed [µg/g]**	**Non-Creatinine Ratioed [ng/mL]**	**Creatinine Ratioed [µg/g]**	**Non-Creatinine Ratioed [ng/mL]**	**Creatinine Ratioed [µg/g]**
All	All	41.2 [1.23]	42.1 [1.18]	3.15 [0.310]	3.60 [0.263]	3.41 [0.184]	3.37 [0.159]	3.43 [0.137]	3.23 [0.103]
Age	18 – 24	24.9 [2.18]	21.0 [1.34]	3.40 [1.79]†	4.06 [1.65]†	3.17 [0.530]	2.28 [0.278]	3.17 [0.225]	2.42 [0.153]
	25 – 34	41.7 [2.32]	35.3 [1.83]	3.82 [0.729]†	3.69 [0.576]†	4.54 [0.697]	3.65 [0.518]	3.60 [0.386]	2.92 [0.265]
	35 – 54	45.8 [2.20]	47.2 [2.20]	2.81 [0.445]	3.07 [0.344]	3.50 [0.297]	3.32 [0.233]	3.38 [0.260]	3.22 [0.178]
	≥55	42.7 [2.26]	54.5 [2.46]	2.94 [0.759]†	4.40 [0.719]†	2.79 [0.435]	3.67 [0.416]	3.55 [0.247]	3.98 [0.235]
Sex	Male	41.8 [1.53]	36.1 [1.22]	3.97 [0.684]	3.48 [0.500]	3.41 [0.189]	3.33 [0.165]	3.52 [0.210]	2.85 [0.156]
	Female	40.5 [1.77]	49.3 [1.87]	2.70 [0.340]	3.69 [0.335]	3.57 [1.16]†	4.30 [1.05]†	3.38 [0.175]	3.48 [0.126]
Race	Non-Hispanic White	45.1 [1.53]	51.5 [1.56]	3.03 [0.327]	3.71 [0.308]	3.33 [0.196]	3.31 [0.176]	3.47 [0.172]	3.48 [0.151]
	Non-Hispanic Black	39.2 [2.54]	27.4 [1.85]	8.17 [2.50]†	4.39 [1.13]†	4.35 [1.01]†	3.85 [0.742]†	3.62 [0.343]	2.76 [0.209]
	Hispanic	27.3 [2.50]	24.1 [2.03]	2.44 [0.996]†	2.45 [0.680]†	3.77 [2.02]†	4.35 [2.27]†	3.18 [0.264]	2.91 [0.162]
	Other Race/Multiracial Ethnicity	27.4 [4.25]	29.8 [3.95]	1.98 [0.679]†	2.14 [0.613]†	4.29 [1.08]†	3.66 [0.988]†	3.51 [0.511]	3.28 [0.375]
Education	<High School/GED	42.1 [2.25]	43.4 [2.38]	3.17 [0.641]†	3.11 [0.464]†	3.06 [0.538]	3.35 [0.358]	3.44 [0.340]	3.24 [0.291]
	HS Diploma	43.9 [2.22]	44.3 [2.19]	3.50 [0.805]†	3.49 [0.593]†	3.38 [0.371]	3.30 [0.337]	3.24 [0.236]	2.84 [0.151]
	<4y College or Associate Degree	39.5 [1.98]	39.5 [1.81]	2.94 [0.553]	3.61 [0.453]	3.59 [0.392]	3.44 [0.289]	3.57 [0.258]	3.22 [0.182]
	Bachelor/Advanced	33.8 [3.13]	39.8 [2.64]	3.15 [0.800]†	4.41 [0.786]†	3.85 [0.560]†	3.38 [0.443]†	3.48 [0.278]	3.59 [0.223]

† Estimate should be interpreted with caution because it has low statistical precision. It is based on a sample size of less than 50, or the coefficient of variation of the estimate is larger than 30%.

Table S17. Sample-weighted geometric means [standard error] by demographic and tobacco user group for PATH Study Wave 1 (2013-2014): Mandelic acid (MADA).

**MADA**		**Every Day Established Combustible Users**		**Every Day Established E-Cigarette Users**		**Every Day Established Smokeless Users**		**Never Users**	
**Predictor**	**Level**	**Non-Creatinine Ratioed [ng/mL]**	**Creatinine Ratioed [µg/g]**	**Non-Creatinine Ratioed [ng/mL]**	**Creatinine Ratioed [µg/g]**	**Non-Creatinine Ratioed [ng/mL]**	**Creatinine Ratioed [µg/g]**	**Non-Creatinine Ratioed [ng/mL]**	**Creatinine Ratioed [µg/g]**
All	All	294 [6.74]	300 [4.14]	124 [8.61]	143 [6.52]	141 [8.51]	140 [6.00]	136 [4.30]	128 [2.97]
Age	18 – 24	253 [10.9]	214 [6.75]	80.4 [34.3]†	96.1 [16.7]†	144 [16.5]	101 [6.84]	138 [6.53]	105 [4.09]
	25 – 34	318 [13.5]	266 [7.90]	152 [20.7]†	147 [13.8]†	180 [17.4]	146 [15.1]	156 [10.7]	124 [5.35]
	35 – 54	315 [12.7]	327 [7.69]	123 [13.8]	142 [11.7]	147 [8.91]	143 [7.44]	134 [8.53]	130 [6.59]
	≥55	264 [12.8]	335 [9.93]	107 [19.0]†	154 [15.7]†	112 [16.4]	145 [12.7]	126 [7.05]	142 [5.48]
Sex	Male	319 [10.3]	275 [5.89]	139 [15.2]	123 [9.62]	143 [9.07]	141 [6.28]	151 [6.89]	123 [5.31]
	Female	269 [8.88]	329 [7.09]	115 [10.7]	157 [11.5]	104 [24.5]†	128 [20.4]†	127 [5.43]	131 [3.77]
Race	Non-Hispanic White	286 [8.62]	327 [5.43]	117 [8.91]	145 [7.62]	141 [9.49]	141 [6.62]	135 [4.96]	135 [4.62]
	Non-Hispanic Black	352 [17.1]	248 [9.93]	256 [49.7]†	137 [20.8]†	137 [23.2]†	98.9 [14.2]†	147 [15.2]	114 [6.40]
	Hispanic	288 [18.5]	250 [10.0]	127 [42.9]†	128 [19.6]†	132 [24.9]†	156 [34.7]†	136 [9.54]	125 [4.69]
	Other Race/Multiracial Ethnicity	219 [16.6]	249 [12.3]	102 [35.8]†	120 [23.9]†	154 [23.1]†	152 [20.2]†	122 [12.6]	114 [6.63]
Education	<High School/GED	306 [10.1]	312 [6.43]	156 [24.6]†	150 [15.0]†	120 [19.2]	127 [7.46]	127 [8.24]	121 [5.97]
	HS Diploma	299 [13.6]	303 [9.12]	125 [20.1]†	125 [11.8]†	162 [10.5]	161 [12.7]	142 [11.0]	125 [6.32]
	<4y College or Associate Degree	296 [10.2]	295 [7.03]	114 [14.5]	144 [10.8]	133 [12.7]	130 [9.60]	129 [5.98]	117 [4.08]
	Bachelor/Advanced	219 [15.2]	263 [13.7]	119 [24.8]†	167 [30.4]†	162 [22.3]†	142 [11.4]†	141 [8.60]	146 [6.14]

† Estimate should be interpreted with caution because it has low statistical precision. It is based on a sample size of less than 50, or the coefficient of variation of the estimate is larger than 30%.

Table S18. Sample-weighted geometric means [standard error] by demographic and tobacco user group for PATH Study Wave 1 (2013-2014): *N*-acetyl-S-(4-hydroxy-2-buten-1-yl)-L-cysteine (t4HBEMA).

t4HBEMA		Every Day Established Combustible Users		Every Day Established E-Cigarette Users		Every Day Established Smokeless Users		Never Users	
**Predictor**	**Level**	**Non-Creatinine Ratioed [ng/mL]**	**Creatinine Ratioed [μg/g]**	**Non-Creatinine Ratioed [ng/mL]**	**Creatinine Ratioed [μg/g]**	**Non-Creatinine Ratioed [ng/mL]**	**Creatinine Ratioed [μg/g]**	**Non-Creatinine Ratioed [ng/mL]**	**Creatinine Ratioed [μg/g]**
All	All	31.9 [0.787]	32.6 [0.708]	4.05 [0.299]	4.58 [0.243]	4.44 [0.200]	4.35 [0.176]	4.72 [0.155]	4.43 [0.104]
Age	18 – 24	21.9 [1.90]	18.4 [1.08]	3.51 [1.33]†	4.19 [1.42]†	4.02 [0.441]	2.84 [0.250]	4.58 [0.212]	3.49 [0.136]
	25 – 34	32.8 [1.56]	27.7 [1.17]	4.74 [0.652]†	4.48 [0.503]†	5.27 [0.489]	4.24 [0.294]	4.93 [0.347]	4.00 [0.237]
	35 – 54	34.6 [1.30]	35.8 [1.33]	3.86 [0.445]	4.22 [0.377]	4.69 [0.290]	4.46 [0.253]	4.59 [0.291]	4.36 [0.169]
	≥55	32.1 [1.51]	41.0 [1.59]	3.68 [0.673]†	5.50 [0.475]†	3.74 [0.313]	4.83 [0.325]	4.84 [0.272]	5.42 [0.251]
Sex	Male	33.0 [1.01]	28.5 [0.659]	4.89 [0.529]	4.24 [0.385]	4.49 [0.213]	4.36 [0.187]	5.19 [0.234]	4.21 [0.185]
	Female	30.8 [1.26]	37.5 [1.29]	3.54 [0.337]	4.85 [0.300]	3.57 [0.804]†	4.30 [0.618]†	4.46 [0.188]	4.57 [0.115]
Race	Non-Hispanic White	33.1 [0.997]	37.8 [0.988]	3.87 [0.294]	4.68 [0.273]	4.37 [0.214]	4.31 [0.185]	4.67 [0.198]	4.68 [0.133]
	Non-Hispanic Black	33.0 [1.72]	23.1 [1.21]	10.1 [3.48]†	5.41 [1.45]†	5.27 [1.42]†	4.67 [0.851]†	5.04 [0.509]	3.84 [0.217]
	Hispanic	26.1 [2.01]	23.0 [1.27]	3.62 [1.50]†	3.62 [0.985]†	4.29 [1.49]†	4.98 [1.99]†	4.54 [0.315]	4.14 [0.183]
	Other Race/Multiracial Ethnicity	23.4 [2.68]	25.7 [2.26]	2.52 [0.953]†	2.71 [0.841]†	5.38 [0.705]†	4.60 [0.708]†	5.02 [0.549]	4.62 [0.391]
Education	<High School/GED	31.9 [1.36]	33.0 [1.33]	4.01 [0.627]†	3.93 [0.567]†	4.49 [0.447]	4.86 [0.450]	4.82 [0.317]	4.53 [0.231]
	HS Diploma	34.2 [1.39]	34.5 [1.23]	4.79 [0.643]†	4.64 [0.491]†	4.47 [0.296]	4.34 [0.260]	4.80 [0.324]	4.20 [0.203]
	<4y College or Associate Degree	30.9 [1.40]	30.9 [1.34]	3.84 [0.567]	4.72 [0.419]	4.35 [0.262]	4.16 [0.214]	4.78 [0.257]	4.29 [0.181]
	Bachelor/Advanced	27.0 [2.06]	31.8 [1.42]	3.48 [0.622]†	4.86 [0.616]†	4.47 [0.621]†	3.93 [0.349]†	4.56 [0.300]	4.71 [0.229]

† Estimate should be interpreted with caution because it has low statistical precision. It is based on a sample size of less than 50, or the coefficient of variation of the estimate is larger than 30%.

Table S19. Sample-weighted geometric means [standard error] by demographic and tobacco user group for PATH Study Wave 1 (2013-2014): Phenylglyoxylic acid (PHGA).

PHGA		Every Day Established Combustible Users		Every Day Established E-Cigarette Users		Every Day Established Smokeless Users		Never Users	
**Predictor**	**Level**	**Non-Creatinine Ratioed [ng/mL]**	**Creatinine Ratioed [µg/g]**	**Non-Creatinine Ratioed [ng/mL]**	**Creatinine Ratioed [µg/g]**	**Non-Creatinine Ratioed [ng/mL]**	**Creatinine Ratioed [µg/g]**	**Non-Creatinine Ratioed [ng/mL]**	**Creatinine Ratioed [µg/g]**
All	All	391 [8.57]	398 [5.73]	189 [13.2]	220 [9.80]	228 [9.24]	223 [7.39]	215 [6.29]	202 [4.26]
Age	18 – 24	360 [17.2]	304 [9.83]	165 [47.8]†	193 [18.1]†	245 [30.9]	172 [11.5]	217 [7.60]	167 [4.13]
	25 – 34	443 [16.7]	369 [14.2]	223 [27.1]†	211 [18.6]†	259 [30.7]	195 [17.8]	233 [14.8]	188 [6.28]
	35 – 54	401 [15.4]	414 [10.8]	194 [20.6]	213 [13.5]	235 [10.1]	225 [6.25]	205 [10.9]	197 [8.65]
	≥55	352 [14.3]	445 [11.7]	149 [30.5]†	256 [34.5]†	198 [24.2]	258 [22.8]	218 [11.0]	240 [8.88]
Sex	Male	426 [11.4]	367 [8.00]	220 [18.9]	197 [13.3]	231 [9.80]	223 [7.64]	236 [12.0]	194 [8.86]
	Female	358 [12.1]	433 [7.90]	171 [15.9]	237 [15.5]	181 [30.4]†	222 [51.5]†	203 [7.23]	207 [4.56]
Race	Non-Hispanic White	390 [10.8]	443 [7.34]	185 [16.0]	231 [11.7]	226 [10.6]	222 [8.20]	219 [8.16]	220 [7.33]
	Non-Hispanic Black	427 [21.6]	297 [10.9]	260 [48.1]†	130 [25.3]†	237 [44.4]†	210 [35.5]†	215 [16.3]	164 [8.02]
	Hispanic	388 [23.0]	341 [13.1]	204 [68.8]†	205 [47.5]†	219 [36.7]†	253 [40.5]†	215 [15.3]	193 [8.92]
	Other Race/Multiracial Ethnicity	304 [22.4]	333 [17.2]	172 [44.5]†	185 [20.3]†	269 [45.4]†	229 [23.4]†	194 [18.0]	182 [8.56]
Education	<High School/GED	401 [13.9]	412 [10.1]	252 [46.7]†	262 [41.5]†	211 [18.2]	227 [15.3]	221 [11.6]	203 [8.67]
	HS Diploma	399 [17.5]	405 [12.7]	192 [25.4]†	195 [19.4]†	251 [18.3]	246 [20.4]	218 [11.8]	194 [7.89]
	<4y College or Associate Degree	396 [11.3]	386 [9.99]	166 [20.2]	206 [11.7]	220 [14.3]	208 [12.1]	208 [11.5]	189 [7.62]
	Bachelor/Advanced	301 [24.9]	360 [15.9]	196 [29.8]†	270 [30.9]†	229 [26.6]†	201 [14.4]†	217 [11.7]	222 [10.0]

† Estimate should be interpreted with caution because it has low statistical precision. It is based on a sample size of less than 50, or the coefficient of variation of the estimate is larger than 30%.

Table S20. Sample-weighted geometric means [standard error] by demographic and tobacco user group for PATH Study Wave 1 (2013-2014): *N*-acetyl-S-(phenyl)-L-cysteine (PHMA).

PMA		Every Day Established Combustible Users		Every Day Established E-Cigarette Users		Every Day Established Smokeless Users		Never Users	
**Predictor**	**Level**	**Non-Creatinine Ratioed [ng/mL]**	**Creatinine Ratioed [µg/g]**	**Non-Creatinine Ratioed [ng/mL]**	**Creatinine Ratioed [µg/g]**	**Non-Creatinine Ratioed [ng/mL]**	**Creatinine Ratioed [µg/g]**	**Non-Creatinine Ratioed [ng/mL]**	**Creatinine Ratioed [µg/g]**
All	All	1.04 [0.024]	1.06 [0.0210]	0.919 [0.060]	1.04 [0.070]	0.985 [0.041]	0.966 [0.048]	1.07 [0.042]	1.01 [0.039]
Age	18 – 24	1.06 [0.053]	0.888 [0.059]	0.665 [0.151]†	0.795 [0.296]†	0.912 [0.105]	0.645 [0.081]	1.03 [0.042]	0.780 [0.031]
	25 – 34	1.04 [0.040]	0.880 [0.034]	0.947 [0.114]†	0.894 [0.105]†	1.09 [0.107]	0.877 [0.105]	1.16 [0.086]	0.938 [0.075]
	35 – 54	1.08 [0.056]	1.12 [0.048]	1.00 [0.109]	1.10 [0.108]	1.07 [0.072]	1.02 [0.064]	1.03 [0.063]	0.982 [0.062]
	≥55	0.962 [0.038]	1.23 [0.043]	0.832 [0.152]†	1.24 [0.173]†	0.819 [0.083]	1.06 [0.107]	1.11 [0.081]	1.24 [0.085]
Sex	Male	1.10 [0.036]	0.951 [0.031]	0.974 [0.118]	0.843 [0.088]	0.995 [0.042]	0.966 [0.051]	1.18 [0.081]	0.957 [0.066]
	Female	0.977 [0.030]	1.19 [0.032]	0.882 [0.073]	1.21 [0.105]	0.799 [0.228]†	0.961 [0.220]†	1.01 [0.044]	1.04 [0.047]
Race	Non-Hispanic White	1.04 [0.030]	1.19 [0.026]	0.878 [0.063]	1.06 [0.079]	0.989 [0.046]	0.975 [0.056]	1.09 [0.059]	1.09 [0.056]
	Non-Hispanic Black	0.969 [0.058]	0.677 [0.040]	1.57 [0.548]†	0.844 [0.257]†	0.840 [0.086]†	0.744 [0.106]†	0.812 [0.051]	0.620 [0.042]
	Hispanic	1.17 [0.084]	1.03 [0.051]	1.10 [0.557]†	1.10 [0.486]†	0.815 [0.253]†	0.942 [0.319]†	1.28 [0.103]	1.17 [0.077]
	Other Race/Multiracial Ethnicity	0.995 [0.070]	1.10 [0.079]	0.810 [0.173]†	0.873 [0.174]†	1.20 [0.113]†	1.02 [0.171]†	0.985 [0.108]	0.905 [0.101]
Education	<High School/GED	1.05 [0.043]	1.09 [0.038]	1.02 [0.136]†	0.997 [0.142]†	0.898 [0.069]	0.971 [0.115]	1.03 [0.071]	0.969 [0.063]
	HS Diploma	0.990 [0.041]	1.000 [0.038]	0.933 [0.145]†	0.903 [0.111]†	0.986 [0.071]	0.959 [0.072]	1.22 [0.110]	1.07 [0.083]
	<4y College or Associate Degree	1.09 [0.046]	1.09 [0.050]	0.861 [0.085]	1.06 [0.118]	1.07 [0.076]	1.02 [0.078]	1.02 [0.053]	0.916 [0.061]
	Bachelor/Advanced	0.946 [0.059]	1.11 [0.057]	0.957 [0.153]†	1.34 [0.217]†	0.976 [0.191]†	0.858 [0.146]†	1.04 [0.077]	1.07 [0.074]

† Estimate should be interpreted with caution because it has low statistical precision. It is based on a sample size of less than 50, or the coefficient of variation of the estimate is larger than 30%.

Table S21. Sample-weighted geometric means [standard error] by demographic and tobacco user group for PATH Study Wave 1 (2013-2014): 2-thioxothiazolidine-4-carboxylic acid (TTCA).

TTCA		Every Day Established Combustible Users		Every Day Established E-Cigarette Users		Every Day Established Smokeless Users		Never Users	
**Predictor**	**Level**	**Non-Creatinine Ratioed [ng/mL]**	**Creatinine Ratioed [µg/g]**	**Non-Creatinine Ratioed [ng/mL]**	**Creatinine Ratioed [µg/g]**	**Non-Creatinine Ratioed [ng/mL]**	**Creatinine Ratioed [µg/g]**	**Non-Creatinine Ratioed [ng/mL]**	**Creatinine Ratioed [µg/g]**
All	All	21.1 [0.521]	21.5 [0.556]	17.1 [1.78]	19.8 [2.12]	18.8 [0.997]	18.1 [1.27]	22.3 [1.30]	20.6 [1.27]
Age	18 – 24	19.5 [1.22]	16.6 [0.808]	14.7 [5.41]	18.6 [8.13]	17.4 [2.52]	12.4 [1.98]	19.7 [1.14]	14.6 [0.922]
	25 – 34	21.9 [1.26]	18.3 [0.952]	18.1 [3.06]	17.4 [2.70]	21.9 [3.71]	16.6 [3.00]	25.1 [2.32]	20.3 [2.19]
	35 – 54	22.9 [1.23]	23.6 [1.18]	15.4 [1.57]	17.2 [1.57]	19.0 [1.57]	17.6 [1.59]	22.3 [2.53]	20.6 [2.37]
	≥55	18.7 [1.03]	23.8 [1.43]	19.7 [5.69]	29.5 [9.64]	17.4 [1.99]	22.0 [3.29]	21.9 [2.22]	24.8 [2.55]
Sex	Male	22.9 [0.930]	19.7 [0.836]	15.2 [1.95]	13.4 [2.16]	19.0 [1.06]	18.1 [1.33]	23.7 [2.08]	19.3 [2.08]
	Female	19.3 [0.644]	23.7 [0.942]	18.7 [2.73]	26.5 [3.07]	15.0 [4.13]	18.1 [6.55]	21.4 [1.58]	21.4 [1.55]
Race	Non-Hispanic White	19.8 [0.652]	22.7 [0.834]	16.4 [1.78]	20.1 [2.34]	18.4 [1.10]	17.8 [1.40]	24.6 [2.19]	24.0 [2.33]
	Non-Hispanic Black	27.6 [2.21]	19.2 [1.70]	33.9 [19.5]	17.1 [8.65]	31.6 [11.1]	23.7 [6.52]	18.0 [1.43]	13.7 [1.20]
	Hispanic	19.5 [1.35]	16.9 [1.23]	19.6 [6.40]	19.6 [3.84]	15.8 [3.56]	18.3 [4.96]	19.4 [1.71]	17.8 [1.51]
	Other Race/Multiracial Ethnicity	22.4 [2.56]	24.5 [2.44]	12.3 [1.62]	16.8 [7.30]	20.1 [3.11]	17.8 [2.52]	23.2 [3.43]	21.5 [4.04]
Education	<High School/GED	21.4 [1.22]	21.9 [1.08]	11.7 [1.28]	11.1 [2.06]	20.2 [2.56]	21.1 [3.61]	18.3 [1.75]	17.1 [1.77]
	HS Diploma	20.7 [0.915]	20.4 [1.08]	17.4 [3.63]	17.3 [4.05]	21.6 [2.90]	20.5 [2.72]	23.7 [3.20]	20.6 [2.90]
	<4y College or Associate Degree	21.8 [1.09]	22.2 [1.10]	17.1 [1.87]	22.1 [2.10]	16.1 [1.34]	14.9 [1.25]	19.2 [1.32]	17.0 [1.22]
	Bachelor/Advanced	18.7 [1.51]	22.4 [1.41]	25.9 [8.40]	37.0 [9.90]	16.2 [2.14]	14.3 [2.31]	26.9 [2.51]	27.2 [2.79]

† Estimate should be interpreted with caution because it has low statistical precision. It is based on a sample size of less than 50, or the coefficient of variation of the estimate is larger than 30%.

Table S22. Sample-weighted creatinine and non-creatinine ratioed percentiles (95% confidence interval) by tobacco user group for PATH Study Wave 1 (2013-2014): 2-Methylhippuric acid (2MHA).

Percentile	Every Day Established Combustible Users		Every Day Established E-Cigarette Users		Every Day Established Smokeless Users		Never Users	
	Non-Creatinine Ratioed [ng/mL]	Creatinine Ratioed [µg/g]	Non-Creatinine Ratioed [ng/mL]	Creatinine Ratioed [µg/g]	Non-Creatinine Ratioed [ng/mL]	Creatinine Ratioed [µg/g]	Non-Creatinine Ratioed [ng/mL]	Creatinine Ratioed [µg/g]
5th	26.0 [21.5,30.5]	29.4 [27.4,31.3]	[<LOD]	4.54 [<LOD,9.11]	[<LOD]	6.13 [4.49,7.78]	[<LOD]	4.47 [3.88,5.06]
10th	36.9 [34.3,39.6]	40.1 [35.9,44.3]	4.88 [1.37,8.40]	9.09 [4.04,14.1]	7.95 [6.20,9.70]	10.7 [7.52,13.8]	5.73 [4.74,6.73]	5.74 [4.84,6.65]
25th	68.8 [62.4,75.1]	74.0 [68.3,79.6]	14.3 [10.2,18.4]	17.3 [11.4,23.3]	18.1 [15.1,21.1]	17.4 [14.5,20.3]	11.9 [10.5,13.3]	11.6 [10.1,13.1]
50th	119 [111,127]	121 [113,129]	27.8 [20.0,35.6]	33.8 [26.3,41.2]	33.5 [28.7,38.3]	29.1 [27.1,31.2]	23.6 [20.7,26.6]	22.1 [20.7,23.5]
75th	196 [184,207]	201 [185,217]	62.9 [48.8,77.0]	59.4 [46.9,71.9]	60.3 [47.9,72.7]	62.4 [51.1,73.7]	43.6 [38.8,48.4]	41.7 [35.7,47.7]
90th	318 [295,341]	295 [273,318]	104 [67.3,140]	119 [72.3,166]	139 [97.9,179]	133 [97.6,168]	90.8 [75.6,106]	81.7 [69.3,94.1]
95th	410 [370,450]	379 [359,399]	138 [94.0,183]	179 [96.6,262]	306 [173,439]	252 [74.0,431]	135 [105,166]	122 [96.0,147]

[<LOD]: Less than the limit of detection

Table S23. Sample-weighted creatinine and non-creatinine ratioed percentiles (95% confidence interval) by tobacco user group for PATH Study Wave 1 (2013-2014): 3-Methylhippuric acid + 4-Methylhippuric acid (34MH).

Percentile	Every Day Established Combustible Users		Every Day Established E-Cigarette Users		Every Day Established Smokeless Users		Never Users	
	Non-Creatinine Ratioed [ng/mL]	Creatinine Ratioed [µg/g]	Non-Creatinine Ratioed [ng/mL]	Creatinine Ratioed [µg/g]	Non-Creatinine Ratioed [ng/mL]	Creatinine Ratioed [µg/g]	Non-Creatinine Ratioed [ng/mL]	Creatinine Ratioed [µg/g]
5th	146 [119,172]	188 [156,220]	30.9 [21.3,40.4]	63.1 [46.7,79.6]	38.6 [30.9,46.3]	54.8 [46.3,63.4]	36.6 [31.9,41.3]	48.6 [46.4,50.8]
10th	228 [203,254]	290 [247,333]	39.2 [29.3,49.0]	72.4 [59.0,85.8]	47.9 [39.9,55.8]	69.7 [62.0,77.4]	49.4 [43.1,55.7]	59.3 [53.7,64.9]
25th	416 [362,469]	534 [501,567]	76.6 [44.5,109]	102 [84.9,120]	87.8 [63.5,112]	98.4 [90.6,106]	86.7 [77.4,96.0]	88.9 [83.3,94.5]
50th	806 [752,859]	830 [786,875]	169 [129,208]	176 [142,209]	178 [155,200]	156 [139,172]	156 [142,171]	134 [124,145]
75th	1.38E+03 [1.30E+03,1.46E+03]	1.30E+03 [1.22E+03,1.39E+03]	342 [202,481]	309 [276,342]	334 [287,381]	299 [250,349]	269 [234,304]	227 [203,250]
90th	2.28E+03 [2.17E+03,2.39E+03]	1.80E+03 [1.70E+03,1.89E+03]	611 [451,772]	564 [297,831]	827 [564,1.09E+03]	564 [452,676]	506 [422,590]	419 [321,517]
95th	3.14E+03 [2.88E+03,3.40E+03]	2.16E+03 [1.99E+03,2.33E+03]	1.14E+03 [444,1.83E+03]	980 [727,1.23E+03]	1.27E+03 [701,1.84E+03]	1.01E+03 [175,1.84E+03]	846 [631,1.06E+03]	735 [519,952]

Table S24. Sample-weighted creatinine and non-creatinine ratioed percentiles (95% confidence interval) by tobacco user group for PATH Study Wave 1 (2013-2014): *N*-acetyl-S-(2-carbamoylethyl)- L-cysteine (2CAEMA).

Percentile	Every Day Established Combustible Users		Every Day Established E-Cigarette Users		Every Day Established Smokeless Users		Never Users	
	**Non-Creatinine Ratioed [ng/mL]**	**Creatinine Ratioed [µg/g]**	**Non-Creatinine Ratioed [ng/mL]**	**Creatinine Ratioed [µg/g]**	**Non-Creatinine Ratioed [ng/mL]**	**Creatinine Ratioed [µg/g]**	**Non-Creatinine Ratioed [ng/mL]**	**Creatinine Ratioed [µg/g]**
5th	34.8 [32.0,37.6]	56.5 [54.2,58.9]	10.1 [5.57,14.7]	24.4 [18.6,30.2]	12.2 [8.79,15.6]	18.0 [14.4,21.5]	10.5 [7.71,13.3]	18.0 [16.4,19.6]
10th	47.4 [43.6,51.2]	70.1 [66.4,73.8]	16.2 [11.0,21.3]	28.7 [25.4,32.0]	17.5 [13.5,21.5]	24.1 [20.5,27.7]	16.1 [13.6,18.5]	20.3 [18.3,22.3]
25th	84.8 [77.1,92.4]	102 [98.5,106]	27.1 [23.1,31.2]	38.6 [33.7,43.6]	31.0 [29.7,32.4]	33.8 [30.3,37.2]	30.4 [27.6,33.2]	30.9 [28.3,33.6]
50th	154 [147,162]	151 [144,157]	53.1 [44.5,61.6]	55.4 [49.7,61.0]	49.1 [45.5,52.8]	47.2 [42.9,51.6]	48.8 [44.6,53.1]	44.2 [41.5,47.0]
75th	266 [243,289]	228 [213,242]	102 [79.1,126]	82.9 [52.1,114]	78.2 [70.3,86.0]	67.0 [62.3,71.7]	81.9 [74.3,89.5]	66.2 [63.3,69.1]
90th	432 [399,465]	325 [308,343]	192 [141,243]	138 [78.9,196]	116 [99.6,133]	85.7 [73.9,97.6]	127 [119,136]	93.3 [83.7,103]
95th	554 [516,593]	403 [351,455]	270 [194,345]	227 [8.93,446]	168 [138,197]	114 [89.8,137]	173 [162,184]	125 [112,138]

Table S25. Sample-weighted creatinine and non-creatinine ratioed percentiles (95% confidence interval) by tobacco user group for PATH Study Wave 1 (2013-2014): *N*-acetyl-S-(N-methylcarbamoyl)- L-cysteine (MCAMA).

Percentile	Every Day Established Combustible Users		Every Day Established E-Cigarette Users		Every Day Established Smokeless Users		Never Users	
	**Non-Creatinine Ratioed [ng/mL]**	**Creatinine Ratioed [µg/g]**	**Non-Creatinine Ratioed [ng/mL]**	**Creatinine Ratioed [µg/g]**	**Non-Creatinine Ratioed [ng/mL]**	**Creatinine Ratioed [µg/g]**	**Non-Creatinine Ratioed [ng/mL]**	**Creatinine Ratioed [µg/g]**
5th	131 [120,142]	162 [152,172]	47.4 [28.4,66.4]	62.9 [48.9,77.0]	32.2 [24.5,39.9]	49.7 [37.9,61.6]	26.2 [21.3,31.1]	31.5 [29.2,33.8]
10th	184 [168,200]	218 [197,239]	56.1 [43.6,68.6]	80.4 [57.1,104]	49.3 [39.7,59.0]	64.1 [59.2,68.9]	37.0 [32.8,41.3]	38.5 [35.0,41.9]
25th	318 [296,340]	356 [336,377]	89.0 [71.3,107]	129 [98.0,160]	84.7 [68.9,101]	90.3 [83.6,97.1]	63.4 [56.9,69.9]	59.7 [52.7,66.8]
50th	552 [518,586]	559 [525,592]	181 [138,225]	194 [150,237]	155 [136,173]	132 [119,145]	112 [100,123]	104 [95.4,113]
75th	928 [873,982]	879 [815,943]	365 [300,430]	328 [283,374]	230 [198,261]	196 [168,224]	194 [177,212]	176 [162,190]
90th	1.42E+03 [1.33E+03,1.50E+03]	1.32E+03 [1.21E+03,1.43E+03]	482 [321,644]	521 [416,626]	326 [296,356]	290 [233,347]	316 [298,335]	268 [240,296]
95th	1.80E+03 [1.62E+03,1.99E+03]	1.55E+03 [1.50E+03,1.61E+03]	668 [495,842]	654 [481,826]	387 [326,448]	360 [313,407]	425 [375,475]	359 [327,390]

Table S26. Sample-weighted creatinine and non-creatinine ratioed percentiles (95% confidence interval) by tobacco user group for PATH Study Wave 1 (2013-2014): *N*-acetyl-S-(benzyl)-L-cysteine (BZMA).

Percentile	Every Day Established Combustible Users		Every Day Established E-Cigarette Users		Every Day Established Smokeless Users		Never Users	
	**Non-Creatinine Ratioed [ng/mL]**	**Creatinine Ratioed [µg/g]**	**Non-Creatinine Ratioed [ng/mL]**	**Creatinine Ratioed [µg/g]**	**Non-Creatinine Ratioed [ng/mL]**	**Creatinine Ratioed [µg/g]**	**Non-Creatinine Ratioed [ng/mL]**	**Creatinine Ratioed [µg/g]**
5th	1.29 [1.08,1.51]	2.19 [2.05,2.33]	1.26 [0.684,1.85]	2.03 [1.57,2.48]	1.34 [1.16,1.51]	1.90 [1.53,2.27]	1.41 [1.23,1.58]	1.98 [1.85,2.10]
10th	1.84 [1.65,2.04]	2.69 [2.55,2.82]	1.84 [1.13,2.56]	2.42 [1.86,2.99]	1.92 [1.62,2.22]	2.36 [2.14,2.59]	2.07 [1.83,2.31]	2.34 [2.12,2.56]
25th	3.30 [3.01,3.59]	3.77 [3.57,3.98]	3.16 [2.29,4.03]	4.05 [3.30,4.80]	3.08 [2.57,3.60]	3.29 [2.99,3.60]	3.49 [3.08,3.91]	3.45 [3.17,3.72]
50th	6.27 [5.94,6.60]	5.88 [5.52,6.25]	5.85 [5.07,6.63]	6.65 [5.39,7.91]	5.45 [4.88,6.01]	5.29 [4.98,5.59]	6.18 [5.54,6.82]	5.48 [5.13,5.84]
75th	11.7 [10.7,12.7]	9.99 [9.07,10.9]	13.3 [9.03,17.6]	11.7 [8.55,14.9]	9.98 [8.39,11.6]	8.52 [8.15,8.89]	11.6 [10.3,12.9]	9.81 [8.94,10.7]
90th	22.0 [19.6,24.4]	18.0 [15.8,20.2]	22.3 [15.1,29.6]	29.6 [17.5,41.7]	15.8 [14.2,17.4]	14.2 [11.1,17.2]	21.0 [17.5,24.4]	17.6 [14.7,20.5]
95th	34.3 [26.1,42.5]	31.3 [23.5,39.1]	44.3 [9.82,78.9]	37.4 [29.7,45.1]	20.4 [16.4,24.3]	18.5 [12.5,24.5]	29.5 [26.3,32.8]	25.5 [20.7,30.3]

Table S27. Sample-weighted creatinine and non-creatinine ratioed percentiles (95% confidence interval) by tobacco user group for PATH Study Wave 1 (2013-2014): *N*-acetyl-S-(2-carboxyethyl)-L-cysteine (2COEMA).

Percentile	Every Day Established Combustible Users		Every Day Established E-Cigarette Users		Every Day Established Smokeless Users		Never Users	
	**Non-Creatinine Ratioed [ng/mL]**	**Creatinine Ratioed [µg/g]**	**Non-Creatinine Ratioed [ng/mL]**	**Creatinine Ratioed [µg/g]**	**Non-Creatinine Ratioed [ng/mL]**	**Creatinine Ratioed [µg/g]**	**Non-Creatinine Ratioed [ng/mL]**	**Creatinine Ratioed [µg/g]**
5th	56.3 [49.9,62.8]	92.4 [83.5,101]	17.7 [11.9,23.5]	42.4 [32.9,51.8]	23.2 [18.3,28.0]	36.8 [32.0,41.7]	23.2 [18.2,28.3]	37.6 [34.2,40.9]
10th	91.2 [78.2,104]	121 [112,131]	26.4 [19.2,33.5]	51.5 [35.9,67.0]	31.1 [23.2,38.9]	44.4 [38.2,50.7]	33.3 [29.1,37.5]	46.4 [42.4,50.3]
25th	168 [154,182]	185 [174,195]	47.2 [32.2,62.2]	71.5 [57.9,85.1]	56.8 [45.1,68.5]	62.9 [56.1,69.6]	63.2 [58.4,68.0]	60.7 [57.5,64.0]
50th	320 [300,340]	303 [289,317]	109 [87.8,129]	109 [91.6,127]	95.4 [82.6,108]	89.1 [80.3,97.8]	103 [94.6,112]	91.2 [87.0,95.4]
75th	549 [514,583]	488 [465,512]	229 [168,289]	168 [139,198]	171 [139,202]	138 [122,154]	174 [160,188]	134 [122,145]
90th	856 [795,918]	719 [654,783]	325 [253,396]	271 [203,339]	287 [227,347]	216 [163,270]	280 [258,302]	206 [189,223]
95th	1.08E+03 [972,1.18E+03]	871 [818,925]	393 [165,620]	299 [233,364]	393 [314,472]	314 [270,358]	366 [328,404]	271 [247,294]

Table S28. Sample-weighted creatinine and non-creatinine ratioed percentiles (95% confidence interval) by tobacco user group for PATH Study Wave 1 (2013-2014): *N*-acetyl-S- (1-cyano-2-hydroxyethyl)-L-cysteine (1CYHEMA).

Percentile	Every Day Established Combustible Users		Every Day Established E-Cigarette Users		Every Day Established Smokeless Users		Never Users	
	**Non-Creatinine Ratioed [ng/mL]**	**Creatinine Ratioed [µg/g]**	**Non-Creatinine Ratioed [ng/mL]**	**Creatinine Ratioed [µg/g]**	**Non-Creatinine Ratioed [ng/mL]**	**Creatinine Ratioed [µg/g]**	**Non-Creatinine Ratioed [ng/mL]**	**Creatinine Ratioed [µg/g]**
5th	2.97 [2.33,3.61]	4.13 [3.25,5.01]	[<LOD]	0.850 [0.749,0.951]	[<LOD]	0.681 [0.611,0.752]	[<LOD]	0.701 [0.633,0.769]
10th	5.36 [4.59,6.12]	7.24 [6.31,8.17]	[<LOD]	0.922 [0.763,1.08]	[<LOD]	0.873 [0.802,0.944]	[<LOD]	0.825 [0.780,0.870]
25th	14.2 [12.6,15.7]	15.9 [14.2,17.5]	[<LOD]	1.30 [1.09,1.51]	[<LOD]	1.09 [0.959,1.22]	[<LOD]	1.10 [1.04,1.16]
50th	31.2 [29.0,33.3]	33.3 [30.8,35.8]	[<LOD]	2.18 [1.84,2.52]	[<LOD]	1.68 [1.53,1.83]	[<LOD]	1.57 [1.47,1.67]
75th	62.1 [57.4,66.8]	57.5 [53.9,61.1]	[<LOD]	5.17 [3.67,6.67]	[<LOD]	2.83 [2.36,3.30]	[<LOD]	2.65 [2.37,2.94]
90th	104 [94.3,113]	84.1 [79.9,88.3]	6.74 [<LOD,14.4]	12.0 [7.12,16.9]	[<LOD]	5.76 [4.26,7.26]	[<LOD]	4.80 [3.84,5.77]
95th	144 [136,153]	106 [96.2,116]	20.3 [8.02,32.6]	17.2 [-0.253,34.6]	[<LOD]	9.21 [6.09,12.3]	[<LOD]	6.86 [5.27,8.46]

[<LOD]: Less than the limit of detection

Table S29. Sample-weighted creatinine and non-creatinine ratioed percentiles (95% confidence interval) by tobacco user group for PATH Study Wave 1 (2013-2014): *N*-acetyl-S-(2-cyanoethyl)-L-cysteine (2CYEMA).

Percentile	Every Day Established Combustible Users		Every Day Established E-Cigarette Users		Every Day Established Smokeless Users		Never Users	
	**Non-Creatinine Ratioed [ng/mL]**	**Creatinine Ratioed [µg/g]**	**Non-Creatinine Ratioed [ng/mL]**	**Creatinine Ratioed [µg/g]**	**Non-Creatinine Ratioed [ng/mL]**	**Creatinine Ratioed [µg/g]**	**Non-Creatinine Ratioed [ng/mL]**	**Creatinine Ratioed [µg/g]**
5th	25.7 [17.8,33.7]	32.1 [29.1,35.1]	[<LOD]	0.932 [0.607,1.26]	[<LOD]	0.451 [0.388,0.514]	[<LOD]	0.403 [0.368,0.437]
10th	48.8 [43.7,53.9]	58.6 [52.8,64.3]	0.636 [0.419,0.852]	1.15 [1.02,1.29]	[<LOD]	0.530 [0.470,0.591]	[<LOD]	0.493 [0.458,0.527]
25th	102 [91.4,112]	114 [104,124]	1.12 [0.878,1.37]	1.47 [1.29,1.65]	0.736 [0.535,0.938]	0.817 [0.719,0.914]	0.718 [0.628,0.807]	0.741 [0.687,0.794]
50th	189 [178,200]	200 [186,215]	2.38 [1.73,3.03]	2.62 [1.90,3.33]	1.43 [1.14,1.72]	1.33 [1.12,1.53]	1.21 [1.13,1.29]	1.12 [1.04,1.19]
75th	322 [306,338]	314 [293,335]	9.62 [0.248,19.0]	11.2 [4.52,17.9]	3.27 [2.71,3.82]	2.71 [2.16,3.27]	2.14 [1.94,2.34]	1.79 [1.63,1.94]
90th	516 [472,561]	456 [431,482]	45.0 [2.58,87.3]	40.0 [-36.1,116]	8.85 [6.18,11.5]	9.21 [5.50,12.9]	3.84 [3.40,4.29]	3.14 [2.77,3.51]
95th	700 [642,759]	594 [565,624]	159 [46.7,272]	117 [72.4,161]	17.5 [11.7,23.3]	18.0 [8.75,27.3]	5.69 [3.06,8.32]	4.97 [3.53,6.41]

[<LOD]: Less than the limit of detection

Table S30. Sample-weighted creatinine and non-creatinine ratioed percentiles (95% confidence interval) by tobacco user group for PATH Study Wave 1 (2013-2014): *N*-acetyl-S-(3,4-dihydroxybutyl)- L-cysteine (34HBMA).

Percentile	Every Day Established Combustible Users		Every Day Established E-Cigarette Users		Every Day Established Smokeless Users		Never Users	
	**Non-Creatinine Ratioed [ng/mL]**	**Creatinine Ratioed [µg/g]**	**Non-Creatinine Ratioed [ng/mL]**	**Creatinine Ratioed [µg/g]**	**Non-Creatinine Ratioed [ng/mL]**	**Creatinine Ratioed [µg/g]**	**Non-Creatinine Ratioed [ng/mL]**	**Creatinine Ratioed [µg/g]**
5th	145 [131,160]	269 [255,282]	81.6 [56.0,107]	224 [212,235]	113 [101,126]	206 [190,222]	103 [87.3,119]	192 [178,207]
10th	193 [179,208]	313 [300,325]	99.0 [70.4,128]	243 [221,265]	157 [127,187]	222 [207,237]	146 [127,166]	220 [211,229]
25th	318 [292,344]	388 [374,402]	186 [117,255]	298 [261,336]	249 [227,271]	281 [265,296]	271 [250,291]	267 [258,276]
50th	550 [523,577]	515 [495,534]	376 [314,437]	373 [355,392]	382 [349,415]	356 [340,373]	405 [381,430]	350 [336,364]
75th	809 [767,850]	671 [650,693]	577 [486,669]	475 [453,498]	580 [538,623]	458 [410,507]	594 [558,631]	447 [422,472]
90th	1.14E+03 [1.08E+03,1.19 E+03]	862 [838,885]	833 [705,961]	624 [545,702]	764 [681,847]	556 [504,609]	785 [714,857]	551 [515,587]
95th	1.36E+03 [1.27E+03,1.45 E+03]	1.03E+03 [970,1.09E+03]	944 [745,1.14E+03]	667 [627,707]	900 [681,1.12E+03]	676 [585,767]	989 [907,1.07E+03]	632 [580,685]

Table S31. Sample-weighted creatinine and non-creatinine ratioed percentiles (95% confidence interval) by tobacco user group for PATH Study Wave 1 (2013-2014): *N*-acetyl-S-(2-carbamoyl-2-hydroxyethyl)-L-cysteine (2CAHEMA).

Percentile	Every Day Established Combustible Users		Every Day Established E-Cigarette Users		Every Day Established Smokeless Users		Never Users	
	**Non-Creatinine Ratioed [ng/mL]**	**Creatinine Ratioed [µg/g]**	**Non-Creatinine Ratioed [ng/mL]**	**Creatinine Ratioed [µg/g]**	**Non-Creatinine Ratioed [ng/mL]**	**Creatinine Ratioed [µg/g]**	**Non-Creatinine Ratioed [ng/mL]**	**Creatinine Ratioed [µg/g]**
5th	[<LOD]	6.83 [6.24,7.42]	[<LOD]	4.04 [3.41,4.67]	[<LOD]	3.83 [3.34,4.32]	[<LOD]	3.57 [3.25,3.88]
10th	[<LOD]	9.01 [8.41,9.61]	[<LOD]	4.77 [4.01,5.53]	[<LOD]	4.55 [3.99,5.11]	[<LOD]	4.11 [3.81,4.40]
25th	9.78 [8.98,10.6]	13.2 [12.7,13.8]	[<LOD]	6.91 [5.85,7.97]	[<LOD]	5.49 [4.93,6.05]	[<LOD]	5.63 [5.28,5.98]
50th	18.9 [17.8,19.9]	19.1 [18.5,19.7]	[<LOD]	10.2 [8.65,11.7]	[<LOD]	8.09 [7.18,9.00]	[<LOD]	8.34 [7.77,8.92]
75th	31.4 [30.0,32.9]	27.1 [25.6,28.7]	13.8 [12.3,15.3]	17.9 [15.5,20.4]	11.9 [10.9,12.9]	12.0 [10.4,13.5]	12.1 [11.6,12.6]	12.2 [11.2,13.2]
90th	50.7 [48.0,53.4]	38.5 [37.0,40.0]	21.8 [14.1,29.4]	32.2 [18.4,45.9]	16.4 [14.5,18.2]	19.1 [17.1,21.1]	18.5 [16.4,20.5]	19.2 [16.7,21.7]
95th	66.1 [61.8,70.4]	47.4 [43.5,51.2]	36.1 [21.4,50.9]	47.2 [27.0,67.4]	19.3 [15.3,23.3]	26.0 [19.9,32.1]	25.6 [23.1,28.1]	24.8 [20.0,29.6]

[<LOD]: less than the limit of detection

Table S32. Sample-weighted creatinine and non-creatinine ratioed percentiles (95% confidence interval) by tobacco user group for PATH Study Wave 1 (2013-2014): *N*-acetyl-S-(2-hydroxyethyl)-L-cysteine (2HEMA).

Percentile	Every Day Established Combustible Users		Every Day Established E-Cigarette Users		Every Day Established Smokeless Users		Never Users	
	Non-Creatinine Ratioed [ng/mL]	Creatinine Ratioed [µg/g]	Non-Creatinine Ratioed [ng/mL]	Creatinine Ratioed [µg/g]	Non-Creatinine Ratioed [ng/mL]	Creatinine Ratioed [µg/g]	Non-Creatinine Ratioed [ng/mL]	Creatinine Ratioed [µg/g]
5th	[<LOD]	0.634 [0.519,0.750]	[<LOD]	0.310 [0.236,0.384]	[<LOD]	0.296 [0.276,0.315]	[<LOD]	0.293 [0.268,0.319]
10th	[<LOD]	0.884 [0.781,0.987]	[<LOD]	0.360 [0.285,0.435]	[<LOD]	0.353 [0.316,0.391]	[<LOD]	0.375 [0.345,0.404]
25th	1.51 [1.35,1.67]	1.68 [1.53,1.84]	[<LOD]	0.666 [0.515,0.817]	[<LOD]	0.530 [0.479,0.581]	[<LOD]	0.537 [0.490,0.585]
50th	2.94 [2.71,3.17]	3.15 [3.01,3.28]	[<LOD]	1.10 [0.903,1.31]	[<LOD]	0.888 [0.751,1.02]	0.849 [0.770,0.927]	0.902 [0.831,0.974]
75th	6.19 [5.56,6.82]	5.78 [5.31,6.25]	1.47 [1.17,1.76]	1.77 [1.53,2.02]	1.34 [1.23,1.44]	1.37 [1.15,1.59]	1.63 [1.45,1.81]	1.54 [1.31,1.78]
90th	11.1 [10.2,12.1]	10.7 [9.59,11.8]	2.44 [1.85,3.03]	3.09 [2.00,4.17]	2.14 [1.88,2.40]	2.24 [1.89,2.58]	3.06 [2.61,3.51]	2.70 [2.23,3.17]
95th	17.2 [14.9,19.5]	14.7 [13.1,16.2]	3.21 [1.25,5.17]	4.16 [3.24,5.08]	2.64 [2.28,3.01]	2.88 [2.52,3.24]	4.48 [3.36,5.60]	4.03 [2.77,5.30]

[<LOD]: less than the limit of detection

Table S33. Sample-weighted creatinine and non-creatinine ratioed percentiles (95% confidence interval) by tobacco user group for PATH Study Wave 1 (2013-2014): *N*-acetyl-S-(2-hydroxypropyl)-L-cysteine (2HPMA).

Percentile	Every Day Established Combustible Users		Every Day Established E-Cigarette Users		Every Day Established Smokeless Users		Never Users	
	**Non-Creatinine Ratioed [ng/mL]**	**Creatinine Ratioed [μg/g]**	**Non-Creatinine Ratioed [ng/mL]**	**Creatinine Ratioed [μg/g]**	**Non-Creatinine Ratioed [ng/mL]**	**Creatinine Ratioed [μg/g]**	**Non-Creatinine Ratioed [ng/mL]**	**Creatinine Ratioed [μg/g]**
5th	16.5 [14.2,18.8]	23.5 [20.8,26.3]	[<LOD]	12.7 [10.3,15.1]	5.64 [2.47,8.82]	10.2 [8.08,12.3]	7.39 [6.30,8.48]	10.7 [10.0,11.5]
10th	23.6 [20.6,26.7]	30.4 [27.6,33.1]	8.84 [4.99,12.7]	14.4 [12.7,16.2]	8.78 [6.79,10.8]	11.8 [10.9,12.6]	10.8 [9.38,12.2]	12.6 [11.6,13.6]
25th	42.3 [38.9,45.7]	49.8 [47.1,52.5]	16.9 [13.5,20.3]	22.2 [18.6,25.8]	15.0 [13.6,16.4]	16.3 [15.1,17.5]	16.8 [14.6,18.9]	17.2 [16.6,17.9]
50th	81.4 [76.0,86.8]	81.2 [77.6,84.9]	36.0 [27.5,44.5]	36.1 [28.1,44.1]	26.8 [23.2,30.4]	22.9 [20.0,25.8]	29.9 [28.1,31.7]	24.8 [23.6,26.1]
75th	146 [136,155]	128 [118,139]	63.5 [46.9,80.1]	55.5 [48.0,63.1]	47.3 [39.2,55.3]	40.8 [35.5,46.1]	53.2 [48.0,58.4]	42.7 [36.6,48.8]
90th	240 [217,262]	202 [185,220]	119 [85.6,152]	143 [53.1,232]	86.0 [69.5,102]	68.8 [58.1,79.5]	105 [81.2,129]	103 [67.5,139]
95th	306 [263,348]	258 [233,284]	174 [33.2,315]	200 [112,289]	141 [52.2,231]	127 [7.49,247]	232 [140,324]	198 [83.2,313]

[<LOD]: Less than the limit of detection

Table S34. Sample-weighted creatinine and non-creatinine ratioed percentiles (95% confidence interval) by tobacco user group for PATH Study Wave 1 (2013-2014): *N*-acetyl-S-(3-hydroxypropyl)-L-cysteine (3HPMA).

Percentile	Every Day Established Combustible Users		Every Day Established E-Cigarette Users		Every Day Established Smokeless Users		Never Users	
	**Non-Creatinine Ratioed [ng/mL]**	**Creatinine Ratioed [μg/g]**	**Non-Creatinine Ratioed [ng/mL]**	**Creatinine Ratioed [μg/g]**	**Non-Creatinine Ratioed [ng/mL]**	**Creatinine Ratioed [μg/g]**	**Non-Creatinine Ratioed [ng/mL]**	**Creatinine Ratioed [μg/g]**
5th	261 [227,296]	295 [246,343]	39.2 [<LOD,82.6]	126 [99.2,154]	65.1 [55.8,74.4]	100 [88.7,112]	63.6 [54.6,72.5]	89.7 [83.9,95.4]
10th	403 [359,446]	472 [427,518]	81.9 [64.5,99.2]	148 [121,175]	82.0 [68.1,96.0]	113 [96.8,129]	93.8 [74.1,114]	109 [101,117]
25th	769 [719,819]	828 [753,902]	149 [105,193]	218 [185,252]	153 [139,167]	165 [145,184]	163 [148,177]	161 [150,173]
50th	1.38E+03 [1.30E+03,1.46E+03]	1.38E+03 [1.30E+03,1.46E+03]	319 [227,411]	333 [289,376]	240 [208,271]	225 [203,247]	270 [246,293]	250 [234,266]
75th	2.34E+03 [2.23E+03,2.44E+03]	2.35E+03 [2.18E+03,2.52E+03]	664 [514,813]	531 [451,612]	431 [389,474]	348 [315,381]	480 [426,533]	403 [353,453]
90th	3.80E+03 [3.50E+03,4.09E+03]	3.34E+03 [3.14E+03,3.55E+03]	1.11E+03 [777,1.45E+03]	809 [612,1.01E+03]	701 [497,906]	597 [463,732]	872 [796,947]	689 [578,800]
95th	4.94E+03 [4.45E+03,5.43E+03]	4.35E+03 [4.01E+03,4.69E+03]	1.54E+03 [1.17E+03,1.91E+03]	1.16E+03 [631,1.68E+03]	1.17E+03 [950,1.39E+03]	810 [627,993]	1.17E+03 [967,1.37E+03]	995 [878,1.11E+03]

[<LOD]: Less than the limit of detection

Table S35. Sample-weighted creatinine and non-creatinine ratioed percentiles (95% confidence interval) by tobacco user group for PATH Study Wave 1 (2013-2014): *N*-acetyl-S-(3-hydroxypropyl-1-methyl)-L-cysteine (3HMPMA).

Percentile	Every Day Established Combustible Users		Every Day Established E-Cigarette Users		Every Day Established Smokeless Users		Never Users	
	**Non-Creatinine Ratioed [ng/mL]**	**Creatinine Ratioed [µg/g]**	**Non-Creatinine Ratioed [ng/mL]**	**Creatinine Ratioed [µg/g]**	**Non-Creatinine Ratioed [ng/mL]**	**Creatinine Ratioed [µg/g]**	**Non-Creatinine Ratioed [ng/mL]**	**Creatinine Ratioed [µg/g]**
5th	578 [498,657]	575 [470,679]	71.8 [52.1,91.6]	196 [161,231]	129 [103,155]	198 [176,219]	123 [99.6,147]	198 [193,203]
10th	821 [720,921]	900 [780,1.02E+03]	111 [67.3,155]	248 [200,296]	182 [148,216]	224 [203,245]	162 [130,194]	223 [210,236]
25th	1.56E+03 [1.44E+03,1.69E+03]	1.77E+03 [1.64E+03,1.90E+03]	234 [172,297]	305 [265,345]	283 [254,311]	295 [277,313]	291 [262,319]	290 [280,300]
50th	2.96E+03 [2.78E+03,3.14E+03]	2.99E+03 [2.82E+03,3.17E+03]	436 [339,533]	407 [340,474]	449 [388,509]	416 [368,465]	471 [445,497]	396 [376,416]
75th	4.93E+03 [4.57E+03,5.30E+03]	4.93E+03 [4.68E+03,5.18E+03]	667 [548,785]	608 [548,669]	702 [648,757]	603 [547,659]	761 [676,847]	606 [561,650]
90th	7.70E+03 [7.18E+03,8.22E+03]	6.68E+03 [6.42E+03,6.95E+03]	1.19E+03 [824,1.55E+03]	933 [560,1.31E+03]	1.09E+03 [973,1.21E+03]	874 [763,985]	1.22E+03 [1.01E+03,1.42E+03]	978 [802,1.16E+03]
95th	9.98E+03 [9.13E+03,1.08E+04]	8.23E+03 [7.49E+03,8.97E+03]	1.57E+03 [1.16E+03,1.99E+03]	1.15E+03 [585,1.71E+03]	1.34E+03 [952,1.72E+03]	1.32E+03 [825,1.81E+03]	1.99E+03 [1.49E+03,2.49E+03]	1.69E+03 [1.17E+03,2.21E+03]

Table S36. Sample-weighted creatinine and non-creatinine ratioed percentiles (95% confidence interval) by tobacco user group for PATH Study Wave 1 (2013-2014): *N*-acetyl-S-(4-hydroxy-2-methyl-2-buten-1-yl)-L-cysteine (4HMBEMA).

Percentile	Every Day Established Combustible Users		Every Day Established E-Cigarette Users		Every Day Established Smokeless Users		Never Users	
	Non-Creatinine Ratioed [ng/mL]	Creatinine Ratioed [µg/g]	Non-Creatinine Ratioed [ng/mL]	Creatinine Ratioed [µg/g]	Non-Creatinine Ratioed [ng/mL]	Creatinine Ratioed [µg/g]	Non-Creatinine Ratioed [ng/mL]	Creatinine Ratioed [µg/g]
5th	5.45 [4.85,6.05]	5.78 [4.73,6.83]	[<LOD]	0.824 [0.614,1.03]	[<LOD]	0.726 [0.530,0.922]	[<LOD]	0.878 [0.741,1.02]
10th	9.70 [8.51,10.9]	12.1 [10.5,13.6]	[<LOD]	1.23 [0.758,1.70]	[<LOD]	1.27 [0.914,1.62]	0.774 [0.608,0.939]	1.13 [1.01,1.25]
25th	22.8 [21.1,24.6]	26.7 [24.4,28.9]	1.35 [0.859,1.84]	1.91 [1.40,2.42]	1.62 [1.02,2.23]	1.97 [1.77,2.18]	1.83 [1.61,2.05]	1.93 [1.77,2.08]
50th	47.5 [42.5,52.5]	48.8 [44.8,52.8]	3.35 [2.18,4.52]	3.55 [3.00,4.11]	3.56 [3.17,3.95]	3.58 [2.97,4.19]	3.58 [3.22,3.95]	3.22 [2.94,3.50]
75th	89.1 [81.0,97.2]	81.6 [77.5,85.7]	6.78 [5.66,7.89]	6.25 [4.79,7.71]	6.58 [5.56,7.61]	5.79 [5.27,6.32]	6.43 [6.07,6.80]	5.04 [4.55,5.54]
90th	143 [132,154]	120 [111,128]	13.5 [2.19,24.8]	11.7 [5.74,17.6]	12.4 [9.18,15.6]	8.92 [7.59,10.3]	11.1 [9.73,12.5]	8.51 [7.54,9.48]
95th	187 [168,207]	158 [141,176]	25.8 [18.9,32.6]	15.9 [12.6,19.2]	18.4 [15.3,21.5]	11.8 [8.37,15.2]	17.6 [14.0,21.2]	11.4 [9.11,13.7]

[<LOD]: less than the limit of detection

Table S37. Sample-weighted creatinine and non-creatinine ratioed percentiles (95% confidence interval) by tobacco user group for PATH Study Wave 1 (2013-2014): Mandelic acid (MADA).

Percentile	Every Day Established Combustible Users		Every Day Established E-Cigarette Users		Every Day Established Smokeless Users		Never Users	
	Non-Creatinine Ratioed [ng/mL]	Creatinine Ratioed [µg/g]	Non-Creatinine Ratioed [ng/mL]	Creatinine Ratioed [µg/g]	Non-Creatinine Ratioed [ng/mL]	Creatinine Ratioed [µg/g]	Non-Creatinine Ratioed [ng/mL]	Creatinine Ratioed [µg/g]
5th	70.9 [61.4,80.5]	114 [108,121]	18.6 [3.34,33.9]	62.9 [47.0,78.7]	36.3 [25.8,46.7]	53.7 [44.0,63.4]	33.3 [24.5,42.1]	55.0 [51.3,58.6]
10th	100 [87.1,113]	142 [135,150]	35.6 [20.6,50.7]	74.2 [54.3,94.1]	55.7 [48.0,63.3]	70.7 [60.6,80.8]	52.4 [43.5,61.3]	67.8 [63.8,71.8]
25th	176 [165,186]	209 [201,216]	74.1 [58.9,89.3]	103 [89.0,117]	94.9 [81.5,108]	97.4 [89.1,106]	91.7 [79.8,104]	90.7 [86.0,95.4]
50th	312 [291,333]	300 [285,315]	137 [109,165]	136 [124,147]	142 [121,163]	134 [123,145]	146 [136,156]	129 [121,137]
75th	510 [477,543]	439 [404,474]	224 [188,260]	192 [164,219]	234 [196,272]	195 [180,211]	222 [210,233]	183 [170,195]
90th	786 [733,839]	599 [560,638]	329 [283,375]	253 [179,328]	306 [283,329]	265 [227,303]	337 [302,371]	243 [222,263]
95th	1.01E+03 [868,1.15E+03]	749 [692,805]	407 [316,497]	349 [242,457]	396 [304,489]	342 [225,458]	429 [374,485]	294 [269,319]

Table S38. Sample-weighted creatinine and non-creatinine ratioed percentiles (95% confidence interval) by tobacco user group for PATH Study Wave 1 (2013-2014): *N*-acetyl-S-(4-hydroxy-2-buten-1-yl)-L-cysteine (t4HBEMA).

Percentile	Every Day Established Combustible Users		Every Day Established E-Cigarette Users		Every Day Established Smokeless Users		Never Users	
	**Non-Creatinine Ratioed [ng/mL]**	**Creatinine Ratioed [µg/g]**	**Non-Creatinine Ratioed [ng/mL]**	**Creatinine Ratioed [µg/g]**	**Non-Creatinine Ratioed [ng/mL]**	**Creatinine Ratioed [µg/g]**	**Non-Creatinine Ratioed [ng/mL]**	**Creatinine Ratioed [µg/g]**
5th	6.20 [5.41,6.99]	7.56 [6.15,8.96]	0.902 [0.500,1.30]	1.77 [1.39,2.15]	1.43 [1.12,1.74]	1.83 [1.56,2.10]	1.19 [1.01,1.37]	1.74 [1.56,1.93]
10th	9.45 [8.44,10.5]	12.1 [11.0,13.2]	1.20 [0.936,1.47]	2.14 [1.88,2.39]	1.65 [1.52,1.79]	2.13 [1.93,2.33]	1.67 [1.54,1.81]	2.14 [2.02,2.27]
25th	18.5 [17.1,19.8]	21.9 [20.6,23.2]	2.35 [1.72,2.99]	2.85 [2.55,3.15]	2.62 [2.13,3.10]	2.92 [2.64,3.20]	2.97 [2.68,3.25]	2.95 [2.82,3.09]
50th	34.3 [32.3,36.4]	34.4 [32.7,36.2]	4.04 [3.16,4.91]	4.07 [3.53,4.61]	4.31 [3.66,4.95]	4.09 [3.68,4.50]	4.84 [4.41,5.26]	4.25 [3.99,4.52]
75th	61.0 [57.6,64.3]	55.5 [52.6,58.5]	7.00 [5.59,8.42]	6.98 [5.85,8.11]	7.58 [6.79,8.36]	6.27 [5.77,6.77]	7.82 [7.25,8.40]	6.22 [5.87,6.58]
90th	92.5 [87.9,97.1]	79.5 [75.8,83.3]	11.9 [7.29,16.5]	9.99 [7.82,12.2]	12.0 [11.1,13.0]	9.92 [8.21,11.6]	12.1 [10.9,13.4]	10.0 [8.66,11.4]
95th	123 [115,132]	96.3 [87.1,106]	20.3 [13.0,27.6]	13.4 [8.62,18.1]	16.5 [14.2,18.8]	12.3 [8.18,16.5]	15.6 [14.2,16.9]	12.0 [10.9,13.0]

Table S39. Sample-weighted creatinine and non-creatinine ratioed percentiles (95% confidence interval) by tobacco user group for PATH Study Wave 1 (2013-2014): Phenylglyoxylic acid (PHGA).

Percentile	Every Day Established Combustible Users		Every Day Established E-Cigarette Users		Every Day Established Smokeless Users		Never Users	
	**Non-Creatinine Ratioed [ng/mL]**	**Creatinine Ratioed [μg/g]**	**Non-Creatinine Ratioed [ng/mL]**	**Creatinine Ratioed [μg/g]**	**Non-Creatinine Ratioed [ng/mL]**	**Creatinine Ratioed [μg/g]**	**Non-Creatinine Ratioed [ng/mL]**	**Creatinine Ratioed [μg/g]**
5th	101 [94.1,108]	155 [147,164]	38.5 [10.6,66.3]	103 [63.9,142]	66.4 [45.8,87.0]	98.4 [84.5,112]	56.9 [48.4,65.4]	92.6 [80.5,105]
10th	138 [122,154]	200 [187,213]	70.3 [56.1,84.6]	120 [108,133]	87.6 [76.1,99.0]	121 [104,137]	86.3 [75.8,96.7]	116 [108,124]
25th	235 [216,254]	288 [276,299]	110 [66.4,154]	149 [130,168]	147 [138,156]	160 [150,171]	149 [136,161]	149 [139,158]
50th	435 [406,465]	412 [398,425]	198 [156,240]	222 [200,245]	225 [190,260]	209 [200,218]	231 [209,253]	203 [195,211]
75th	672 [632,712]	569 [543,596]	323 [264,382]	298 [259,336]	351 [309,392]	297 [276,318]	336 [315,357]	275 [261,290]
90th	975 [918,1.03E+03]	780 [735,826]	491 [394,587]	420 [378,461]	514 [445,583]	409 [369,450]	482 [443,520]	351 [329,374]
95th	1.27E+03 [1.18E+03,1.36E+03]	912 [855,969]	564 [485,643]	499 [194,805]	630 [525,735]	458 [396,519]	585 [519,651]	433 [376,491]

Table S40. Sample-weighted creatinine and non-creatinine ratioed percentiles (95% confidence interval) by tobacco user group for PATH Study Wave 1 (2013-2014): *N*-acetyl-S-(phenyl)-L-cysteine (PHMA).

Percentile	Every Day Established Combustible Users		Every Day Established E-Cigarette Users		Every Day Established Smokeless Users		Never Users	
	**Non-Creatinine Ratioed [ng/mL]**	**Creatinine Ratioed [µg/g]**	**Non-Creatinine Ratioed [ng/mL]**	**Creatinine Ratioed [µg/g]**	**Non-Creatinine Ratioed [ng/mL]**	**Creatinine Ratioed [µg/g]**	**Non-Creatinine Ratioed [ng/mL]**	**Creatinine Ratioed [µg/g]**
5th	[<LOD]	0.321 [0.281,0.361]	[<LOD]	0.250 [0.173,0.328]	[<LOD]	0.296 [0.225,0.368]	[<LOD]	0.272 [0.252,0.293]
10th	[<LOD]	0.415 [0.377,0.454]	[<LOD]	0.342 [0.180,0.503]	[<LOD]	0.378 [0.351,0.405]	[<LOD]	0.354 [0.316,0.391]
25th	[<LOD]	0.649 [0.608,0.691]	[<LOD]	0.657 [0.549,0.765]	[<LOD]	0.522 [0.452,0.591]	[<LOD]	0.554 [0.496,0.612]
50th	0.982 [0.907,1.06]	1.08 [1.02,1.14]	0.798 [0.587,1.01]	1.02 [0.865,1.17]	0.901 [0.723,1.08]	0.907 [0.778,1.04]	1.00 [0.908,1.10]	0.993 [0.906,1.08]
75th	1.77 [1.63,1.91]	1.74 [1.62,1.85]	1.55 [1.26,1.85]	1.80 [1.35,2.24]	1.70 [1.42,1.98]	1.62 [1.40,1.84]	1.94 [1.70,2.17]	1.77 [1.56,1.97]
90th	3.10 [2.77,3.43]	2.58 [2.42,2.75]	2.74 [2.41,3.08]	2.73 [2.21,3.26]	3.28 [2.79,3.77]	2.71 [2.10,3.32]	3.45 [3.17,3.74]	3.03 [2.77,3.29]
95th	4.47 [3.57,5.37]	3.42 [3.13,3.70]	3.90 [2.68,5.12]	3.28 [2.57,3.99]	4.31 [3.74,4.89]	3.59 [3.04,4.14]	4.41 [3.84,4.98]	3.93 [3.39,4.47]

[<LOD]: less than the limit of detection

Table S41. Sample-weighted creatinine and non-creatinine ratioed percentiles (95% confidence interval) by tobacco user group for PATH Study Wave 1 (2013-2014): 2-thioxothiazolidine-4-carboxylic acid (TTCA).

Percentile	Every Day Established Combustible Users		Every Day Established E-Cigarette Users		Every Day Established Smokeless Users		Never Users	
	Non-Creatinine Ratioed [ng/mL]	Creatinine Ratioed [µg/g]	Non-Creatinine Ratioed [ng/mL]	Creatinine Ratioed [µg/g]	Non-Creatinine Ratioed [ng/mL]	Creatinine Ratioed [µg/g]	Non-Creatinine Ratioed [ng/mL]	Creatinine Ratioed [µg/g]
5th	[<LOD]	6.41 [5.97,6.85]	[<LOD]	4.70 [2.91,6.48]	[<LOD]	4.68 [3.78,5.59]	[<LOD]	4.57 [4.23,4.91]
10th	[<LOD]	7.74 [7.29,8.20]	[<LOD]	6.15 [4.33,7.97]	[<LOD]	5.76 [4.96,6.56]	[<LOD]	5.38 [4.68,6.07]
25th	[<LOD]	11.4 [10.8,12.0]	[<LOD]	10.5 [8.55,12.4]	[<LOD]	8.68 [7.54,9.83]	[<LOD]	8.36 [7.12,9.60]
50th	17.6 [16.4,18.8]	18.9 [17.4,20.3]	13.1 [11.6,14.5]	16.9 [12.7,21.2]	14.8 [13.5,16.1]	13.4 [11.6,15.1]	15.9 [14.4,17.4]	18.1 [15.6,20.5]
75th	32.2 [28.4,35.9]	34.7 [32.4,37.0]	23.2 [11.3,35.1]	31.9 [21.2,42.5]	27.7 [21.2,34.1]	30.6 [24.7,36.4]	41.1 [28.8,53.4]	38.0 [31.0,45.0]
90th	76.9 [60.5,93.3]	69.9 [61.1,78.7]	66.2 [43.1,89.3]	69.1 [44.7,93.6]	61.5 [38.7,84.2]	79.6 [50.2,109]	107 [79.4,135]	119 [78.7,160]
95th	130 [110,149]	112 [94.7,130]	137 [29.4,245]	102 [23.0,181]	163 [54.2,273]	142 [51.2,232]	188 [38.9,336]	245 [124,366]

[<LOD]: less than the limit of detection

Table S42. Sample-weighted multivariate regression modeling of predictor variables for urinary N-acetyl-S-(2-carboxyethyl)-L-cysteine (2COEMA). (N=4,983).

Predictor	Level	N	% (SE)	Coefficient [95% CI]	p-Value
Intercept				0.352 [0.0529, 0.651]	
Creatinine, urine (Ln)	Coefficient			0.890 [0.839, 0.942]	<0.0001
Sex	Male	2448	42.9 (0.820)	Ref.	
	Female	2535	57.1 (0.820)	0.0870 [7.77E-03, 0.166]	0.0317
Age Group	18-24	1307	14.5 (0.512)	-0.186 [-0.282, -0.090]	0.0002
	25-34	966	18.9 (0.978)	Ref.	
	35-54	1704	37.6 (1.32)	0.114 [3.67E-03, 0.225]	0.0430
	≥55	1006	28.9 (1.00)	0.275 [0.169, 0.381]	<0.0001
Race/Ethnicity	Non-Hispanic White	3052	60.8 (1.39)	Ref.	
	Non-Hispanic Black	841	14.8 (0.913)	9.60E-03 [-0.0746, 0.094]	0.8214
	Hispanic	722	17.2 (1.04)	-0.162 [-0.261, -0.063]	0.0016
	Other Race/Multiracial	368	7.21 (0.654)	-0.0319 [-0.135, 0.071]	0.5399
Education Level	<High School/GED	1323	20.3 (0.815)	0.0643 [-0.022, 0.151]	0.1432
	HS Diploma	1310	26.8 (1.23)	Ref.	
	<4y College/Associate	1766	29.7 (1.21)	-0.0307 [-0.124, 0.062]	0.5131
	Bachelors	584	23.3 (1.43)	-0.0642 [-0.181, 0.053]	0.2785
Tobacco User Group	Smokers	2998	29.3 (0.748)	1.12 [1.05, 1.18]	<0.0001
	ENDS Users	138	0.994 (0.097)	0.145 [9.15E-03, 0.280]	0.0367
	Smokeless Users	337	2.48 (0.179)	-5.31E-03 [-0.120, 0.109]	0.9270
	Never Users	1510	67.3 (0.819)	Ref.	

Table S43. Sample-weighted multivariate regression modeling of predictor variables for urinary N-acetyl-S-(2-cyanoethyl)-L-cysteine (2CYEMA). (N=5,245).

Predictor	Level	N	% (SE)	Coefficient [95% CI]	p-Value
Intercept				-2.99 [-3.38, -2.59]	
Creatinine, urine (Ln)	Coefficient			0.726 [0.657, 0.794]	<0.0001
Sex	Male	2578	43.2 (0.769)	Ref.	
	Female	2667	56.8 (0.769)	1.99E-03 [-0.103, 0.107]	0.9702
Age Group	18-24	1358	14.2 (0.504)	-0.167 [-0.339, 5.60E-03]	0.0578
	25-34	1013	18.7 (0.984)	Ref.	
	35-54	1815	37.8 (1.29)	0.0325 [-0.0953, 0.160]	0.6153
	≥55	1059	29.3 (1.07)	0.0367 [-0.123, 0.196]	0.6490
Race/Ethnicity	Non-Hispanic White	3226	61.3 (1.36)	Ref.	
	Non-Hispanic Black	873	14.5 (0.865)	0.0564 [-0.103, 0.215]	0.4830
	Hispanic	760	17.1 (1.02)	-0.147 [-0.319, 0.025]	0.0932
	Other Race/Multiracial	386	7.15 (0.647)	0.0286 [-0.155, 0.212]	0.7583
Education Level	<High School/GED	1395	20.3 (0.794)	0.0668 [-0.121, 0.255]	0.4827
	HS Diploma	1375	27.3 (1.28)	Ref.	
	<4y College/Associate	1856	29.4 (1.16)	-0.118 [-0.261, 0.025]	0.1035
	Bachelors	619	23.0 (1.39)	-0.184 [-0.348, -0.020]	0.0282
Tobacco User Group	Smokers	3180	29.7 (0.710)	4.82 [4.71, 4.93]	<0.0001
	ENDS Users	149	1.03 (0.093)	1.17 [0.928, 1.42]	<0.0001
	Smokeless Users	353	2.48 (0.182)	0.245 [0.0685, 0.421]	<0.0001
	Never Users	1563	66.8 (0.781)	Ref.	

Table S44. Sample-weighted multivariate regression modeling of predictor variables for urinary N-acetyl-S-(3-hydroxypropyl)-L-cysteine (3HPMA). (N=5,199).

Predictor	Level	N	% (SE)	Coefficient [95% CI]	p-Value
Intercept				1.94 [1.61, 2.27]	
Creatinine, urine (Ln)	Coefficient			0.789 [0.726, 0.851]	<0.0001
Sex	Male	2549	43.1 (0.766)	Ref.	
	Female	2650	56.9 (0.766)	0.0334 [-0.041, 0.107]	0.3734
Age Group	18-24	1352	14.3 (0.504)	-0.191 [-0.296, -0.087]	0.0004
	25-34	1001	18.7 (0.988)	Ref.	
	35-54	1791	37.7 (1.29)	0.147 [0.025, 0.268]	0.0185
	≥55	1055	29.4 (1.07)	0.159 [0.068, 0.250]	0.0008
Race/Ethnicity	Non-Hispanic White	3189	61.2 (1.37)	Ref.	
	Non-Hispanic Black	871	14.5 (0.869)	-0.295 [-0.408, -0.182]	<0.0001
	Hispanic	756	17.1 (1.03)	-0.0964 [-0.224, 0.031]	0.1376
	Other Race/Multiracial	383	7.16 (0.650)	0.253 [0.078, 0.428]	0.0050
Education Level	<High School/GED	1382	20.3 (0.799)	8.42E-03 [-0.0988, 0.116]	0.8766
	HS Diploma	1364	27.3 (1.29)	Ref.	
	<4y College/Associate	1837	29.3 (1.16)	-0.0622 [-0.173, 0.048]	0.2662
	Bachelors	616	23.0 (1.39)	-0.0926 [-0.231, 0.046]	0.1868
Tobacco User Group	Smokers	3143	29.5 (0.694)	1.58 [1.51, 1.66]	<0.0001
	ENDS Users	147	1.01 (0.098)	0.221 [0.0748, 0.366]	0.0034
	Smokeless Users	346	2.45 (0.179)	-0.115 [-0.217, -0.0127]	0.0280
	Never Users	1563	67.0 (0.769)	Ref.	

Table S45**.** Sample-weighted multivariate regression modeling of predictor variables for urinary N-acetyl-S-(3-hydroxypropyl-1-methyl)-L-cysteine (3HMPMA). (N=5,245).

Predictor	Level	N	% (SE)	Coefficient [95% CI]	p-Value
Intercept				2.11 [1.82, 2.40]	
Creatinine, urine (Ln)	Coefficient			0.842 [0.788, 0.896]	<.0001
Sex	Male	2578	43.2 (0.769)	Ref.	
	Female	2667	56.8 (0.769)	0.121 [0.036, 0.207]	0.0060
Age Group	18-24	1358	14.2 (0.504)	-0.218 [-0.301, -0.134]	<0.0001
	25-34	1013	18.7 (0.984)	Ref.	
	35-54	1815	37.8 (1.29)	0.157 [0.059, 0.256]	0.0020
	≥55	1059	29.3 (1.07)	0.304 [0.212, 0.396]	<0.0001
Race/Ethnicity	Non-Hispanic White	3226	61.3 (1.36)	Ref.	
	Non-Hispanic Black	873	14.5 (0.865)	-0.327 [-0.425, -0.230]	<0.0001
	Hispanic	760	17.1 (1.02)	-0.196 [-0.326, -0.066]	0.0035
	Other Race/Multiracial	386	7.15 (0.647)	-0.0465 [-0.225, 0.132]	0.6054
Education Level	<High School/GED	1395	20.3 (0.794)	0.0874 [-0.036, 0.211]	0.1636
	HS Diploma	1375	27.3 (1.28)	Ref.	
	<4y College/Associate	1856	29.4 (1.16)	-0.0156 [-0.121, 0.090]	0.7701
	Bachelors	619	23.0 (1.39)	0.0240 [-0.103, 0.151]	0.7084
Tobacco User Group	Smokers	3180	29.7 (0.710)	1.80 [1.73, 1.88]	<0.0001
	ENDS Users	149	1.03 (0.093)	-0.106 [-0.276, 0.0636]	0.2181
	Smokeless Users	353	2.48 (0.182)	-0.0454 [-0.144, 0.0532]	0.3631
	Never Users	1563	66.8 (0.781)	Ref.	

Table S46. Sample-weighted multivariate regression modeling of predictor variables for urinary N-acetyl-S-(4-hydroxy-2-methyl-2-buten-1-yl)-L-cysteine (4HMBEMA). (N=5,221).

Predictor	Level	N	% (SE)	Coefficient [95% CI]	p-Value
Intercept				-3.40 [-3.72, -3.08]	
Creatinine, urine (Ln)	Coefficient			0.962 [0.905, 1.02]	<0.0001
Sex	Male	2562	43.2 (0.764)	Ref.	
	Female	2659	56.8 (0.764)	0.208 [0.116, 0.299]	<0.0001
Age Group	18-24	1347	14.2 (0.515)	-0.268 [-0.424, -0.112]	0.0009
	25-34	1008	18.7 (0.975)	Ref.	
	35-54	1809	37.8 (1.30)	0.122 [-0.020, 0.265]	0.0908
	≥55	1057	29.4 (1.07)	0.286 [0.143, 0.430]	0.0001
Race/Ethnicity	Non-Hispanic White	3212	61.4 (1.34)	Ref.	
	Non-Hispanic Black	870	14.5 (0.871)	-0.332 [-0.447, -0.216]	<0.0001
	Hispanic	756	17.1 (1.02)	-0.294 [-0.417, -0.171]	<0.0001
	Other Race/Multiracial	383	7.02 (0.642)	-0.112 [-0.307, 0.083]	0.2564
Education Level	<High School/GED	1390	20.3 (0.793)	0.0855 [-0.051, 0.222]	0.2181
	HS Diploma	1368	27.3 (1.29)	Ref.	
	<4y College/Associate	1846	29.3 (1.16)	0.0424 [-0.079, 0.164]	0.4890
	Bachelors	617	23.0 (1.40)	0.0442 [-0.097, 0.185]	0.5353
Tobacco User Group	Smokers	3176	29.9 (0.714)	2.56 [2.47, 2.64]	<0.0001
	ENDS Users	148	1.02 (0.095)	0.0197 [-0.158, 0.197]	0.8264
	Smokeless Users	348	2.47 (0.181)	0.0425 [-0.0969, 0.182]	0.5468
	Never Users	1549	66.7 (0.784)	Ref.	

Table S47. Sample-weighted multivariate regression modeling of predictor variables for urinary N-acetyl-S-(4-hydroxy-2-buten-1-yl)-L-cysteine (t4HBEMA). (N=5,244).

Predictor	Level	N	% (SE)	Coefficient [95% CI]	p-Value
Intercept				-2.48 [-2.72, -2.23]	
Creatinine, urine (Ln)	Coefficient			0.851 [0.808, 0.894]	<0.0001
Sex	Male	2577	43.2 (0.769)	Ref.	
	Female	2667	56.8 (0.769)	0.0933 [0.032, 0.155]	0.0033
Age Group	18-24	1358	14.2 (0.504)	-0.200 [-0.300, -0.101]	0.0001
	25-34	1013	18.7 (0.985)	Ref.	
	35-54	1814	37.8 (1.29)	0.105 [0.014, 0.196]	0.0234
	≥55	1059	29.3 (1.07)	0.250 [0.154, 0.347]	<0.0001
Race/Ethnicity	Non-Hispanic White	3226	61.3 (1.36)	Ref.	
	Non-Hispanic Black	873	14.5 (0.865)	-0.234 [-0.334, -0.134]	<0.0001
	Hispanic	759	17.1 (1.02)	-0.185 [-0.280, -0.089]	0.0002
	Other Race/Multiracial	386	7.15 (0.647)	-0.0375 [-0.184, 0.109]	0.6125
Education Level	<High School/GED	1395	20.3 (0.794)	0.0254 [-0.072, 0.122]	0.6041
	HS Diploma	1374	27.3 (1.28)	Ref.	
	<4y College/Associate	1856	29.4 (1.16)	-0.0186 [-0.111, 0.074]	0.6906
	Bachelors	619	23.0 (1.39)	-0.0362 [-0.147, 0.074]	0.5171
Tobacco User Group	Smokers	3180	29.7 (0.709)	1.97 [1.90, 2.03]	<0.0001
	ENDS Users	149	1.03 (0.093)	-0.0493 [-0.170, 0.0710]	0.4184
	Smokeless Users	352	2.48 (0.182)	-0.0571 [-0.156, 0.0419]	0.2553
	Never Users	1563	66.8 (0.781)	Ref.	
